# ATP synthase activity boosts membrane proton acceptance and lateral diffusion

**DOI:** 10.1073/pnas.2510444123

**Published:** 2026-03-03

**Authors:** Hendrik Flegel, Ambili Ramanthrikkovil Variyam, Nadav Amdursky, Claudia Steinem

**Affiliations:** ^a^Faculty of Chemistry, Institute of Organic and Biomolecular Chemistry, University of Göttingen, Göttingen 37077, Germany; ^b^Schulich Faculty of Chemistry, Technion–Israel Institute of Technology, Haifa 3200003, Israel; ^c^Chemistry, School of Mathematical and Physical Sciences, University of Sheffield, Sheffield S3 7HF, United Kingdom

**Keywords:** energy conversion, HPTS, proton transfer, time-correlated single photon counting, unilamellar vesicles

## Abstract

In most organisms, ATP synthesis is powered by the proton motive force (pmf) and catalyzed by ATP synthase. While the chemiosmotic theory originally proposed a “delocalized coupling” between proton pumps and consumers, growing evidence implicates the membrane in mediating localized proton transfer (PT). To directly track ultrafast PT at the membrane surface as a function of ATP synthase activity, we developed an in vitro system. We tethered a light-activated excited-state photoacid to the bilayer of unilamellar vesicles to confine PT to the membrane interface and co-reconstituted a thermophilic *Bacillus* TF_O_F_1_ ATP synthase. Using steady-state and time-resolved fluorescence spectroscopy, we quantified PT and lateral proton diffusion from the anchored photoacid under conditions with non-ATP- and ATP-producing enzymes. Our results show that the membrane accepts protons at its interface and that PT is enhanced only when ATP synthase is active. A comparison with soluble photoacid positioned near the membrane shows that protons consumed by ATP synthase do not equilibrate with the bulk aqueous phase. Instead, they are transferred directly along the two-dimensional membrane interface to the enzyme. This localized coupling can explain how ATP synthesis can proceed even when the apparent bulk pmf seems insufficient. Our results refine the proton translocation during ATP synthesis by revealing that the membrane itself is an active participant in PT, thereby strengthening the case for localized proton coupling in bioenergetics.

## Significance

The proton motive force (pmf) drives adenosine triphosphate (ATP) synthesis in living organisms. However, there are instances where the pmf appears to be insufficient to fuel ATP production. Indirect studies have suggested that lateral proton coupling between proton pumps (pmf generators) and ATP synthase (the proton consumer) may help overcome this challenge, but direct proof has been lacking. To address this knowledge gap, we co-reconstituted a membrane-anchored photoacid with an active F_O_F_1_ ATP synthase in unilamellar vesicles. If the synthase produced ATP, we observed ultrafast proton transfer from the photoacid and membrane-confined proton diffusion. This finding suggests that protons are locally coupled between the proton sources and consumers and may explain why an apparently insufficient pmf still drives ATP-synthesis.

Energy conversion in biological systems is the hallmark of life. Its pivotal intermediate state is an electrochemical gradient established out of equilibrium across bioenergetic membranes, in which protons play the major role. According to Mitchell’s classical chemiosmotic theory (1), the stored electrochemical potential energy, the proton motive force (pmf), is composed of a difference in proton concentration and electrical potential across the membrane. The original chemiosmotic hypothesis assumed a constant pmf between two bulk phases across the coupling membrane. The pmf is generated and maintained by membrane-bound proton pumps and used, for example, for ATP production by the F_O_F_1_ ATP synthase (2). However, several lines of evidence challenged the idea of a constant pmf between two bulk phases. While it was long believed that the pmf is constant in time and space along the membrane, there is growing evidence pointing toward a rich spatiotemporal dynamical behavior of the pmf (3–6). Moreover, several studies hypothesized that protons that are ejected from the primary proton pumps are first transferred laterally along the membrane and do not quickly equilibrate with protons of the bulk solution (7–13). The pioneering work of Heberle et al. (14) demonstrated that protons released upon illumination of the light-driven pump bacteriorhodopsin are transferred significantly faster along the membrane surface than from the surface to bulk water. Subsequent experimental, computational, and theoretical studies supported this notion (11, 15–17). Protons were shown to rapidly move along the lipid membrane with lateral proton diffusivity nearly matching that of bulk water (12, 13, 18, 19). Membrane surface groups are thought to be involved in lateral proton diffusion (PD), which would compete kinetically with transfer via bulk water (20). An energetic barrier across the bulk water-membrane interface created by oriented water molecules can affect proton activity at this interface (9, 10, 21, 22). The equilibration time between bulk water and the membrane is in the order of seconds (23). Suppose the average distance between the proton release site (producer/source) and the consumption site (consumer/sink) is below a certain value *L*_0_, which defines the coupling distance between surface and bulk protons, the equilibration time is too short, and the protons cannot equilibrate with the bulk.

The lateral proton transfer (PT) between a producer and a consumer appears to be of utmost importance for the proper functioning of the ATP synthase (20). Toth et al. (24) determined the pH gradient within mitochondria of respiring cells and found only a small proton gradient. The measured pH values barely permitted ATP synthesis in a reconstituted system with yeast F_O_F_1_ ATP synthase. However, when the ATP synthase was co-reconstituted with an active proton-translocating cytochrome oxidase, ATP synthesis readily occurred at the measured, physiological pH values. These results suggest kinetic coupling between proton pumping and ATP synthesis. Along the same lines, the conundrum of how ATP synthases of alkaliphilic bacteria can overcome the bioenergetic challenges of achieving robust H^+^-coupled ATP synthesis at external pH values >10 might be solved (25). At such pH values, the pmf is too low to account for the observed ATP synthesis. However, in the case of lateral kinetic coupling, an efficient connection between proton release sites (proton pumps) and consumption sites (ATP synthase) (24, 26) can be reached, and a pH-independent, faster-than-the-diffusion-limit proton translocation across the enzyme (27). Despite this indirect evidence of lateral kinetic coupling, its direct observation is still missing.

**Fig. 1.**
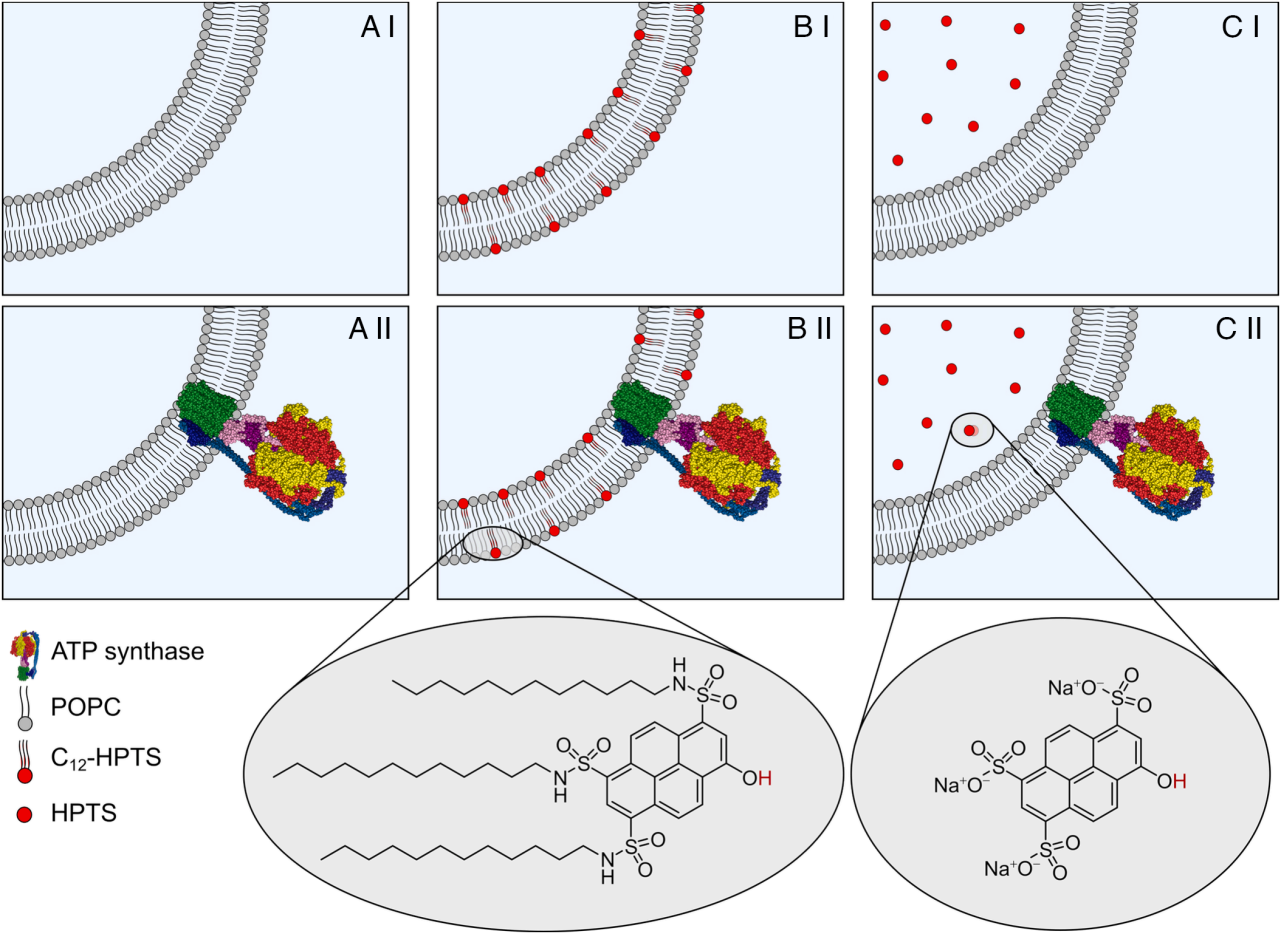
Schematic illustration of the model system. Illustration of part of a large unilamellar vesicle (LUV) and proteo-LUV (pLUV) composed of POPC. (*I*) depict the LUV parts without and (*II*) with embedded TF_O_F_1_ ATP synthase. (*A*) POPC (p)LUV, (*B*) POPC (p)LUV doped with the membrane-tethered photoacid C_12_-HPTS, and (*C*) POPC (p)LUV filled with water-soluble HPTS. The chemical structures of C_12_-HPTS and HPTS are shown below.

To analyse, with very high time resolution, whether producers and consumers of the pmf are laterally and kinetically coupled, we made use of an excited-state molecular photoacid probe (termed C_12_-HPTS (HPTS = 8-Hydroxypyrene-1,3,6-trisulfonate), Fig. 1) that is tethered to a lipid bilayer and can transiently release a proton close to the membrane upon excitation (28–32). In these experiments, the probe is excited and undergoes excited-state proton transfer (ESPT), whereas the membrane surface acts as the proton acceptor. Using ultrafast time-resolved measurements, the time-dependent emission of the membrane-embedded probe provides information about proton dissociation, proton recombination (with the deprotonated probe), and PD. Following the ESPT process, there are two competing processes: proton escape and recombination. Both processes depend on the dimensionality of the PD around the probe. Following the time-dependent emission thus allows the determination of the PT rate and the dimensionality of the PD (33), which is indicative of the coupling of the PT to the membrane.

We compared the PT rates and the dimensionality of PD from the C_12_-HPTS photoacid embedded in the membrane of unilamellar vesicles composed of 1-palmitoyl-2-oleoyl-*sn*-glycero-3-phosphocholine (POPC) in the absence and presence of an active TF_O_F_1_ ATP synthase from a thermophilic *Bacillus* strain (Fig. 1). The TF_O_F_1_ ATP synthase is activated by creating a pmf across the membrane, which is established by a bulk solution pH gradient and not by the light-triggered photoacid. The photoacid C_12_-HPTS serves as a reporter for PT and PD processes occurring at the membrane interface and the possible escape of protons to the bulk. In the presence of the ATP-producing TF_O_F_1_ ATP synthase acting as the proton consumer, an unexpectedly fast PT rate was observed, and the dimensionality of the PD was reduced to a value of 2. In contrast to membrane-embedded C_12_-HPTS, which releases its proton close to the membrane surface upon excitation, soluble HPTS in the lumen of the vesicles did not transfer its proton as quickly as observed for C_12_-HPTS when the TF_O_F_1_ ATP synthase was active.

## Results

### Co-Reconstitution of C_12_-HPTS and the ATP Synthase in POPC-LUVs.

We co-reconstituted the thermophilic *Bacillus* PS3 TF_O_F_1_ ATP synthase and the membrane-anchored photoacid C_12_-HPTS into POPC Large Unilamellar Vesicles (LUVs) to explore how the enzyme affects the PT from C_12_-HPTS and the subsequent PD. To rule out that C_12_-HPTS influences the reconstitution efficiency *R*_eff_ of the ATP synthase, we compared *R*_eff_ of POPC LUVs lacking C_12_-HPTS with POPC LUVs containing C_12_-HPTS. A nominal protein-to-lipid ratio of 1:20,000 was chosen, and the POPC LUVs were doped with 1 mol% C_12_-HPTS. From density gradient centrifugation analyses (*SI Appendix*, Fig. S1), we calculated a reconstitution efficiency of *R*_eff_ *=* 75 ± 12% (*N* = 28) for the ATP synthase in pure POPC vesicles (A.II, Fig. 1). Almost the same value of *R*_eff_ *=* 73 ± 11% (*N* = 41) was obtained in the presence of the membrane-tethered photoacid C_12_-HPTS (B.II, Fig. 1). The reconstitution efficiencies translate into protein-to-lipid ratios of *p*/*l* = 1:27,000 ± 10,000 (*A.II*, *N* = 28), and *p*/*l* = 1:25,000 ± 11,000 (B.II, *N* = 39) (Table 1). These results demonstrate that the amphiphilic C_12_-HPTS, reconstituted into POPC vesicles, does not affect the protein reconstitution efficiency.

**Table 1.** Comparison of the data obtained for protein-containing LUVs (pLUVs) composed of POPC (A.II), POPC/C_12_-HPTS (B.II) and soluble HPTS encapsulated in pLUVs (C.II)

Structure	*R*_eff_ (%)	*p*/*l*	2*r*_pLUVs_ (nm)	*N*_protein_	χ (%)
A.II	75 ± 12	1:27,000 ± 10,000	145 ± 17	10 ± 5	83 ± 2
B.II	73 ± 11	1:25,000 ± 11,000	133 ± 27	8 ± 4	85 ± 5
C.II	78 ± 12	1:21,000 ± 7,000	139 ± 21	9 ± 3	78

The reconstitution efficiency *R*_eff_, the protein-to-lipid-ratio *p*/*l*, the diameter of the pLUVs 2*r*_pLUVs_, the number of proteins per vesicle *N*_protein,_ and the protein fraction χ oriented with its F_1_ subunit facing the vesicle outside are summarized. The values represent mean ± SD.

As the vesicle size may influence the acidification process of the vesicle lumen, we next determined the LUV diameters using dynamic light scattering. Within the error margins, the LUV diameters were the same for structures A.II and B.II (Table 1). LUVs without reconstituted protein showed diameters of about 130 nm (*SI Appendix*, Table S1), in agreement with literature values (34). The trend to slightly larger values found for protein-containing LUVs (pLUVs) was attributed to the large TF_O_F_1_ ATP synthase with an F_1_-subunit that extends approximately 10 nm out of the membrane plane (35). We estimated the number of proteins per vesicle (*N*_protein_) using the pLUV diameter and the protein-to-lipid ratio, assuming an area for a POPC lipid of 0.63 nm^2^ (36) and one for the ATP synthase of 20 nm^2^ (35) (*SI Appendix*). The values were almost the same for structure A.II and B.II, i.e., *N*_protein_ = 10 ± 5 (A.II, *N* = 27) and 8 ± 4 (B.II, *N* = 34) (Table 1).

**Fig. 2.**
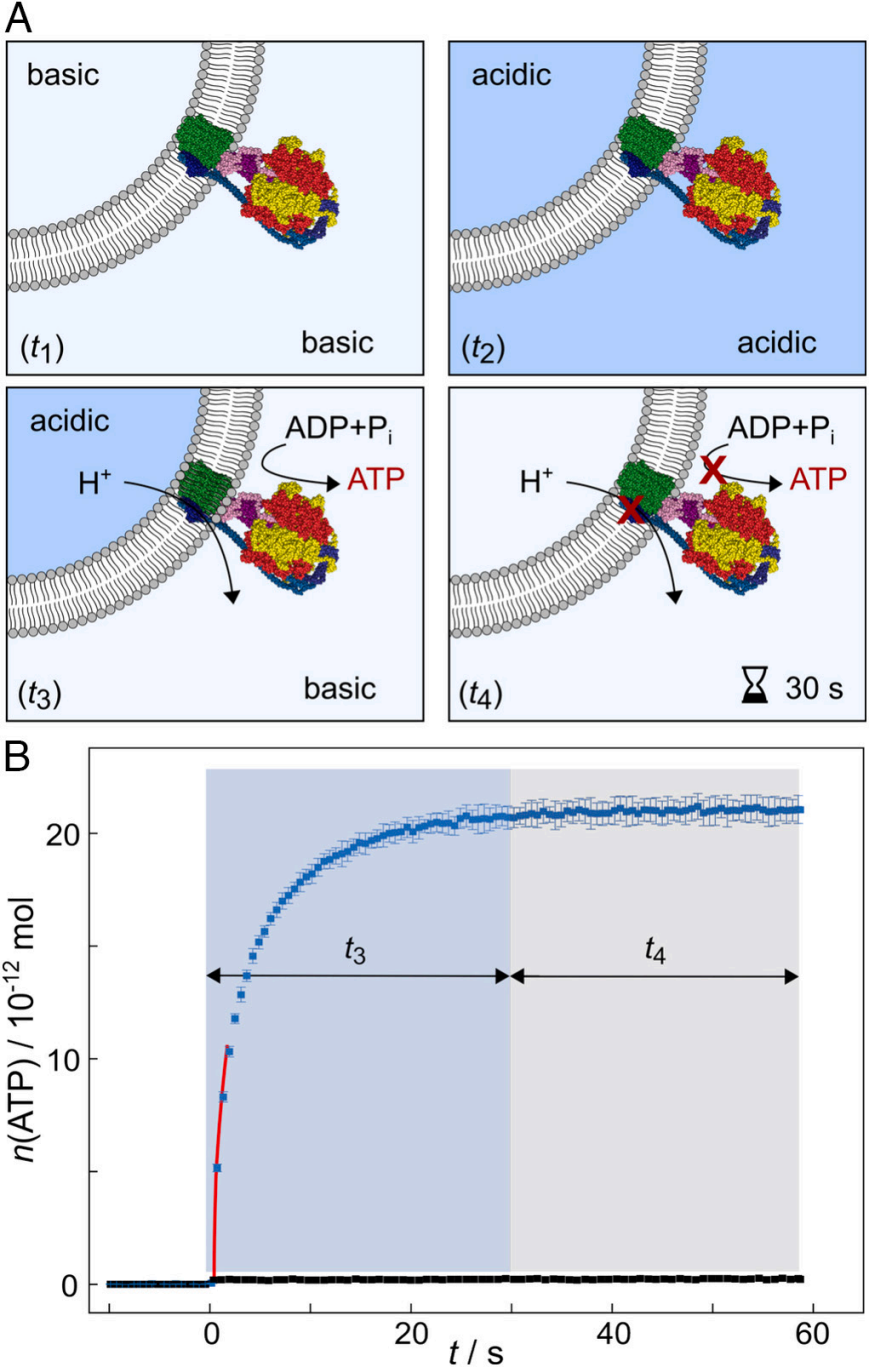
ATP synthesis catalysed by the TF_O_F_1_ ATP synthase. (*A*) Schematic illustration of the acidification procedure of the vesicle lumen before applying a pH gradient that activates the enzyme. pLUVs prepared at pH 8.0 (time window *t*_1_) are acidified (*t*_2_). Buffer exchange to a basic pH establishes the proton gradient, initiating proton translocation, which is mediated by the TF_O_F_1_ ATP synthase and leads to ATP synthesis (*t*_3_). After approximately 30 s, the pmf vanishes, and the protein becomes inactive again (*t*_4_). (*B*) Characteristic time trace of ATP production as a function of time *t* for pLUVs (blue squares) and LUVs lacking the enzyme as a control (black squares). ATP production is observed for approximately 30 s (*t*_3_, blue box) before it ceases (*t*_4_, gray box), as indicated by a constant luminescence signal. The red solid line is the result of extracting $v_{cat}$ from the curve.

*N*_protein_ does not report on the orientation of the protein. Hence, we next analyzed the orientation of the reconstituted ATP synthase. We determined the orientation χ of reconstituted ATP synthase in POPC and POPC/C_12_-HPTS LUVs (*SI Appendix*, Fig. S2). Values of χ = 83 ± 2% (A.II, *N* = 2), and χ = 85 ± 5% (B.II, *N* = 2) (Table 1) demonstrate that more than 80% of the TF_O_F_1_ ATP synthase molecules are oriented with the F_O_ subunit pointing to the outside of the vesicles. The obtained values agree well with those reported in the literature (37). As the pmf is established in the vesicle system, with a more acidic pH in the vesicle lumen (Fig. 2), only those enzymes with the F_1_ subunit pointing to the outside of the vesicles are activated by the pmf. Overall, our results show that the co-reconstitution of C_12_-HPTS into pLUVs does not alter the vesicle structure, protein number, and orientation, which is a prerequisite for a quantitative analysis of the PT kinetics and PD dimensionality.

### Quantification of the ATP Synthase Activity.

Next, we addressed whether the TF_O_F_1_ ATP synthase is equally active in the absence and presence of C_12_-HPTS without illuminating the photoacid, i.e., the C_12_-HPTS does not serve as a proton source. Moreover, we defined the time window in which the enzyme produces ATP using the pmf. We activated the protein by imposing a transmembrane pH gradient of ΔpH = 4.1 (Fig. 2*A*) and monitored ATP production over time using a standard chemiluminescence assay to obtain *n*_ATP_(*t*) (Fig. 2*B*). Establishing the gradient at *t* = 0 s activated the enzyme, marked by the onset of ATP synthesis. An increase in the amount of ATP is observed for ~30 s (time window *t*_3_) until the luminescence signal becomes saturated, indicating a constant ATP concentration (*t*_4_) (Fig. 2*B*). The time trace reports that the ATP synthase is active for ~30 s under the given conditions. We quantified the enzyme’s activity by calculating the turnover number (TON). The TON is defined as (Eq. **1**):

[1] $$\text{TON}=\frac{v_{\text{cat}}}{c_{\text{protein}}},$$

with:

$$v_{\mathrm{cat}}={\left.\frac{dn_{\mathrm{ATP}}(t)}{dt}\right|}_{t=0},$$

$v_{cat}$ is determined from the initial slope of the *n*_ATP_(*t*) curve after protein activation. With the concentration of reconstituted protein *c*_protein_, as determined by density gradient centrifugation (*SI Appendix*), the TON was calculated, reporting on the number of ATP molecules produced per second per ATP synthase. From *N* ≥ 5 independent experiments, we obtained a TON = 8.4 ± 3.1 s−^1^ (A.II, *N* = 20) and a TON = 8.3 ± 3.1 s^-1^ (B.II, *N* = 28), showing that the activity of the TF_O_F_1_ ATP synthase is not influenced by the presence of C_12_-HPTS in the membrane.

### Characteristics of the Light-Triggered Probe C_12_-HPTS.

Following the validation of the ATP synthase activity, we used structure B.II to determine the PT and PD parameters of C_12_-HPTS as a function of ATP synthase activity. C_12_-HPTS is a Brønsted photoacid that becomes acidic only in its excited state due to the large difference in the p*K_a_* values between its ground and excited state (38). The p*K_a_* values of C_12_-HPTS in POPC LUVs and pLUVs were determined to be 7.5 for the ground state and 0.7 for the excited state (*SI Appendix*, Fig. S3 and Table S2). Of note, it is important to emphasize that the protons released from the 1 mol% membrane-tethered photoacid are not the source of the pmf and do not activate the TF_O_F_1_ ATP synthase (*SI Appendix*, Fig. S4). C_12_-HPTS serves as a spectroscopic probe for following fast (ns) PT events from the photoacid on the membrane surface and proton recombination processes, which are sensitive to the PD around the photoacid. Here, we used this photoacid to explore how the activity of the ATP synthase, which occurs on the millisecond-to-second timescale, influences the PT process of the photoacid at the membrane interface. With its three carbon chains, C_12_-HPTS is known to be well integrated into the membrane. Molecular dynamics simulations have shown that the molecule exposes its acidic OH-group toward the water-membrane interface, near the location of the phosphate groups (29).

While exciting the protonated photoacid (ROH), the excited photoacid (ROH^*^) undergoes an excited state photocycle (Eq. **2**):

[2] $$\mathrm{ROH}\overset{h\nu }{\to }{\mathrm{ROH}}^{*}\underset{\mathrm{Rec}.}{\overset{\mathrm{ESPT}}{⇌}}{\mathrm{RO}}^{-*}+H^{+}.$$

Rec. is the proton recombination process with the excited deprotonated species (RO^−*^). The excited probe will deprotonate only in the presence of a proton acceptor. The more efficient the acceptor, the faster the ESPT process. The proton recombination process is directly related to the diffusion of protons away from the photoacid, which is also sensitive to the dimensionality of the PD process. The faster the diffusion away from the excited photoacid (RO^−*^), the lower the proton recombination rate. For C_12_-HPTS embedded in a membrane, the lipid headgroups influence the ESPT process. POPC accepts protons from the probe less efficiently than pure phosphatidic acid (29, 30). In addition, the dimensionality *d* of PD in POPC membranes is fractal, 2 < *d* < 3, indicating that protons can escape from the surface of the membrane to the bulk.

### Steady-State Fluorescence Measurements of C_12_-HPTS in the Presence of Active ATP Synthase.

Owing to the different emission wavelengths of ROH^*^ and RO^−*^, the ratio of the two fluorescence emission maxima in the steady-state spectra reports on their equilibrium concentration ratio, which is equal to (Eq. **3**):

[3] $$\frac{[{\mathrm{RO}}^{-*}]}{\left[{\mathrm{ROH}}^{*}\right]}=\frac{k_{\mathrm{PT}}}{k_{\mathrm{rec}}+k_{\mathrm{rx}}}.$$

**Fig. 3.**
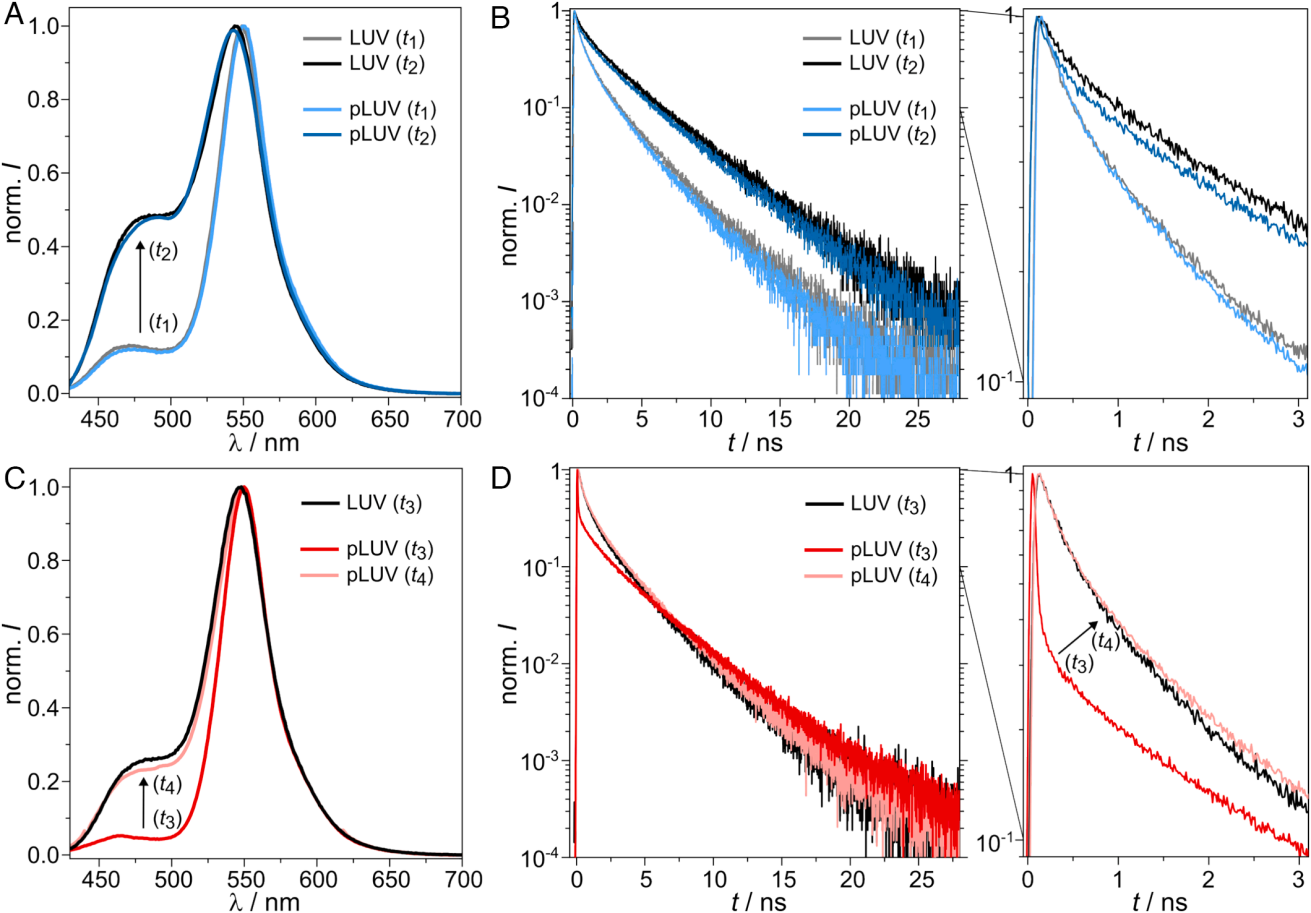
Spectroscopic measurements of C_12_-HPTS in pLUVs. (*A*) Steady-state fluorescence of pLUVs (light blue) during *t*_1_ (cf. Fig. 2) and pLUVs (blue) in phase *t*_2_. LUVs without protein (gray and black) for *t*_1_ and *t*_2_ are shown for comparison. (*B*) Time-resolved fluorescence of LUVs (gray) and pLUVs (light blue) in *t*_1_ as well as LUVs (black) and pLUVs (blue) in *t*_2_. The decay curve of the first 3 ns is shown in a separate zoom-in. (*C*) Steady-state fluorescence of LUVs (black) and pLUVs (red) in the presence of an active protein (*t*_3_) and pLUVs (light red) in the presence of an inactive protein (*t*_4_). (*D*) Time-resolved fluorescence of LUVs (black) and pLUVs (red) in the presence of an active protein (*t*_3_) and pLUVs (light red) in the presence of an inactive protein (*t*_4_). The decay curve of the first 3 ns is shown in a separate zoom-in.

*k*_PT_ is the ESPT rate for the formation of RO^−*^, *k*_rec_ is the proton recombination rate, and *k*_rx_ is the deactivation decay rate of RO^−*^, which will be calculated using time-resolved fluorescence (vide infra). As also inferred from Eq. **2**, C_12_-HPTS must be excited at its protonated (ROH) state to be used as a PT probe (38). Since the ground state p*K_a_* of C_12_-HPTS in POPC membranes is 7.5 (*SI Appendix*, Table S2), we used an acidic buffer with a pH of 4.7. The non-acidic buffer was adjusted to pH = 7.4, resulting in a ΔpH = 2.7. We first recorded steady-state fluorescence emission spectra (Fig. 3*A*) of C_12_-HPTS in POPC LUVs (B.I) and pLUVs (B.II) with symmetric pH conditions of pH 7.4 (time window *t*_1_, Fig. 2*A*) and symmetric pH conditions of pH 4.7 (*t*_2_, Fig. 2*A*). Under both conditions, no pmf is established, and thus, the ATP synthase is inactive. As shown in Fig. 3*A*, the steady-state fluorescence spectra are nearly identical between LUVs with the C_12_-HPTS in the presence and absence of the ATP synthase. As expected, the [RO^−*^]/[ROH^*^] ratio given by the ratio of the two emission maxima is smaller at low pH values (*t*_2_). The larger proton concentration (low pH) acts both to slow the ESPT rate and, more substantially, to increase the proton recombination rate (29–31, 38). These results validate that, under the conditions where the ATP synthase is inactive, the presence of the transmembrane enzyme does not change the PT processes. We then investigated the PT and PD properties after enzyme activation. Upon activating the ATP synthase by creating the pmf (Fig. 2*A*, *t*_3_), we observed a dramatic change in the steady-state fluorescence of the probe in pLUVs during *t*_3_ compared to the LUVs lacking the enzyme (Fig. 3*C*). Immediately after establishing the pmf, the ROH^*^ band nearly completely disappears, resulting in a large [RO^−*^]/[ROH^*^] = 18.6 ± 2.6 (*N* = 4) (Table 2). From the time-dependent measurements of the ATP production, we know that the ATP synthase is active for ~30 s (Fig. 2*B*) and loses activity after this time. Consequently, measuring a second steady-state fluorescence spectrum, taken 2 min after the pH switch, results in [RO^−*^]/[ROH^*^] = 4.1 ± 0.4 (*N* = 4) for pLUVs with inactive enzyme (*t*_4_). This is an increase in the ROH^*^ band to a level similar to that of LUVs without the enzyme ([RO^−*^]/[ROH^*^] = 4.3 ± 0.4, *N* = 4). Such nullification of the ROH^*^ band (a large [RO^−*^]/[ROH^*^]) in the presence of an active ATP synthase was never observed with C_12_-HPTS, regardless of the membrane’s nature, pH, or temperature (29, 30). It can result from a super-fast ESPT process from the probe to the surface of the membrane, a drastic decrease in the recombination process, with some effect of PD dimensionality. To resolve the contribution of each parameter and to understand these intriguing results, we performed time-resolved measurements.

**Table 2.** Comparison of the extracted and fitted parameters of the [RO^−*^]/[ROH^*^] ratio, *k*_PT_, *k*_rec_, and dimensionality for the control LUVs and the pLUVs in the active phase of the enzyme (*t*_3_) and upon its inactivation (*t*_4_)

	[RO-*]/[ROH*]	*k*_PT_ (ns^-1^)	*k*_rec_ (ns^-1^)	*d*
LUVs (*t*_3_)	4.3 ± 0.4	2.5 ± 0.3	0.4 ± 0.07	2.7 ± 0.2
pLUVs (*t*_3_)	18.6 ± 2.6	15.9 ± 2.3	0.7 ± 0.12	1.8 ± 0.3
pLUVs (*t*_4_)	4.1 ± 0.4	2.7 ± 0.4	0.5 ± 0.06	2.5 ± 0.1

The values represent mean ± SD (*N* = 4)

### Time-Resolved Fluorescence Measurements of C_12_-HPTS in the Presence of Active ATP Synthase.

Ultrafast time-resolved fluorescence measurements allow the differentiation between different processes at different time scales following the excitation of ROH. The initial step is ESPT from the tethered probe to the membrane surface (Eq. **2**), observed as the initial decay of ROH^*^. This step depends on the membrane surface’s ability to accept protons and occurs over timescales from a few to hundreds of picoseconds. From an experimental perspective, *k*_PT_ can be extracted directly from these early timescales (33).

Following the ESPT process, two competing processes occur: proton recombination and proton escape. Proton recombination (Eq. **2**) can be followed at time scales of >0.5 ns. Proton escape refers to the “disappearance” of the proton from the vicinity of the probe (RO^−*^) either to the bulk or to a different proton sink. Importantly, both processes depend on the dimensionality of PD around the probe and other diffusion-related parameters. In line with the steady-state measurements, the time-resolved fluorescence curves before establishing the pmf are identical in the presence and absence of the enzyme (Fig. 3*B*). Also in agreement with the steady-state measurements, only when the ATP synthase produces ATP (*t*_3_), a dramatic change in the fluorescence decay of the probe was observed compared to the control (Fig. 3*D*). This change is evident in both the early decay part and the long-lived fluorescence tail. Measuring the fluorescence decay of C_12_-HPTS 2 min after the pH switch (*t*_4_) results in a nearly identical curve compared to the control sample without the enzyme (Fig. 3*D*). During the ATP production phase of the ATP synthase, the initial fluorescence decay of the C_12_-HPTS probe is extremely fast, much faster than what was observed with any reconstituted membrane previously. Fitting this first initial decay (*SI Appendix*, Fig. S5) results in *k*_PT_ = 15.9 ± 2.3 ns^−1^ (*N* = 4) (Table 2). For comparison, the *k*_PT_-value of C_12_-HPTS in POPC LUVs is 2.5 ± 0.3 ns^−1^ (*N* = 4), and that of C_12_-HPTS in pLUVs when the ATP synthase is inactive (*t*_4_) is 2.7 ± 0.4 ns^−1^ (*N* = 4). The very large *k*_PT_-value of C_12_-HPTS in pLUVs containing active ATP synthase was unexpected, since it was even larger than the *k*_PT_-values of HPTS in bulk water (*k*_PT_ of ~9 ns^−1^) (29, 39), which usually represents the upper limit for PT rates, as water is a great proton acceptor. This finding highlights the fundamental role of an active ATP synthase as an extremely efficient proton acceptor for protons originating from the membrane.

The fluorescence decay of ROH^*^ at longer time scales enables the determination of PD-related properties of the geminate proton. As demonstrated by earlier works of Agmon et al. (40, 41) and in the recent work of Stuchebrukhov et al. (33), which simplifies Agmon’s model, the long-lived fluorescent tail of ROH^*^ can be used to extract the PD dimensionality of the geminate proton. These models are based on kinetic diffusion models for the “cloud” of the geminate proton around the excited photoacid following the ESPT process, while considering the geminate recombination process between the released proton and the deprotonated photoacid. Both models can be applied to solvated photoacids or photoacids near surfaces (as discussed in this work). According to the models, the fluorescent tail of ROH^*^ measured in a time-resolved fluorescence experiment and corrected for the radiative lifetime of the photoacid exhibits a power-law decay, which indicates the dimensionality (*d*) of the PD process (Eq. **4**):

[4] $$p_{{\mathrm{ROH}}^{*}}\left(t\right)=\frac{1}{1+(k_{d}^{\prime }t{)}^{d/2}},$$

$p_{{ROH}^{*}}\left(t\right)$ is the ROH^*^ population (without radiative decay), which is related to the TCSPC signal I(t) via: $p_{{ROH}^{*}}\left(t\right)\propto I\left(t\right)e^{\left(t/\tau \right)}$, where τ is the pure radiative decay of C_12_-HPTS (5.6 ns). $k_{d}^{\prime }$ is a complex effective rate of proton dissociation that embodies several PT and PD parameters (Eq. **5**):

[5] $$k_{d}^{\prime }=\frac{k_{\mathrm{PT}}}{B^{2/d}(k_{\mathrm{PT}}\tau_{0}{)}^{1-2/d}}.$$

The PT rate constant *k*_PT_ was determined from the initial decay. *B* is a Boltzmann factor: $B=e^{-\frac{V}{k_{B}T}}$, where *T* is the temperature and the potential $V=-\frac{ze^{2}}{a_{0}\varepsilon }$ depends on the charge (*z*) of the excited photoacid proton donor (*z* = 1 for C_12_-HPTS), the molecular dimension (*a*_0_) of the photoacid donor (*a*_0_ = 6 to 7 Å for C_12_-HPTS), and the medium dielectric constant (ε). While the latter is known for water (ε = 78), it is considerably lower on the surface of lipid membranes by a factor of at least 2 (42). While in water, the Boltzmann factor is ~ 3, on the surface of membranes, we used a value of ~5. The timescale $\tau_{0}$ is defined as: $\tau_{0}=a_{0}^{2}/D$, where *D* is the PD coefficient. In water ($D∼9\cdot 10^{-5}$ cm^2^ s^−1^), $\tau_{0}$ is 40 ps.

By fitting the described model to the $p_{{ROH}^{*}}\left(t\right)$ curve, we can extract the dimensionality from the slope of the long-time asymptotic (*SI Appendix*, Fig. S5). We found that the dimensionality on the surface of the control POPC LUVs is *d* = 2.7 ± 0.2 (*N* = 4) (Table 2). This fractal dimensionality indicates that upon the transient release of the proton from C_12_-HPTS on the surface of the membrane, some of the protons diffuse laterally (2D) while others diffuse toward the bulk water (perpendicular to the membrane, 3D). However, when the ATP synthase produces ATP, the dimensionality of PD from the probe is *d* = 1.8 ± 0.3 (*N* = 4). This value shows that the activity of the ATP synthase results in a pure two-dimensional PD on the membrane surface for protons that are released from C_12_-HPTS. The activity of the ATP synthase prevents the protons from escaping from the membrane surface to the bulk. The magnitude of $p_{{ROH}^{*}}\left(t\right)$ is determined by both *B* and $\tau_{0}$. While using a *B*-factor of ~5, the fit (*SI Appendix*, Fig. S5) results in $\tau_{0}$ values of 300 ± 100 ps for POPC LUVs and 18 ± 10 ps (*N* = 4) for pLUVs with an active ATP synthase (*t*_3_). Since $\tau_{0}$ is inversely related to *D*, we can conclude a dramatic change not only in the dimensionality of PD upon the activation of the ATP synthase but also in the PD coefficient on the surface of the membrane, resulting in more than one order of magnitude increase when the enzyme is active. Since the dielectric constant on the surface of the membrane is unknown, we will refrain from giving an exact value of *D*.

Knowing the *k*_PT_ values and the stationary [RO^−*^]/[ROH^*^] further allows calculating the proton recombination rate *k*_rec_ (Eq. **3**), while taking the deactivation decay rate *k*_rx_ of RO^−*^, which is the same for all samples (0.2 ns^−1^, *SI Appendix*, Fig. S6). The proton recombination rates were calculated to be *k*_rec_ = 0.7 ns^−1^ for pLUVs with an active ATP synthase and 0.4 ns^−1^ for the control POPC LUVs (which is the same order of magnitude as the value for pLUVs with inactive enzymes), respectively (Table 2). The relatively similar proton recombination rate between membranes with an active enzyme and membranes without the enzyme (or an inactive enzyme) implies that the large difference observed in the steady-state measurements is determined primarily by the PT from the probe to the surface of the membrane (Eq. **3**). As we showed in a previous study with pH-dependent measurements of the PT dynamics from the C_12_-HPTS probe within vesicles (30), the recombination process is less sensitive to the pH of the solution, thus indicating that the geminate proton from the membrane surface is the main one recombining with RO^−*^.

### Inhibition of the TF_O_F_1_-ATP Synthase Activity.

**Fig. 4.**
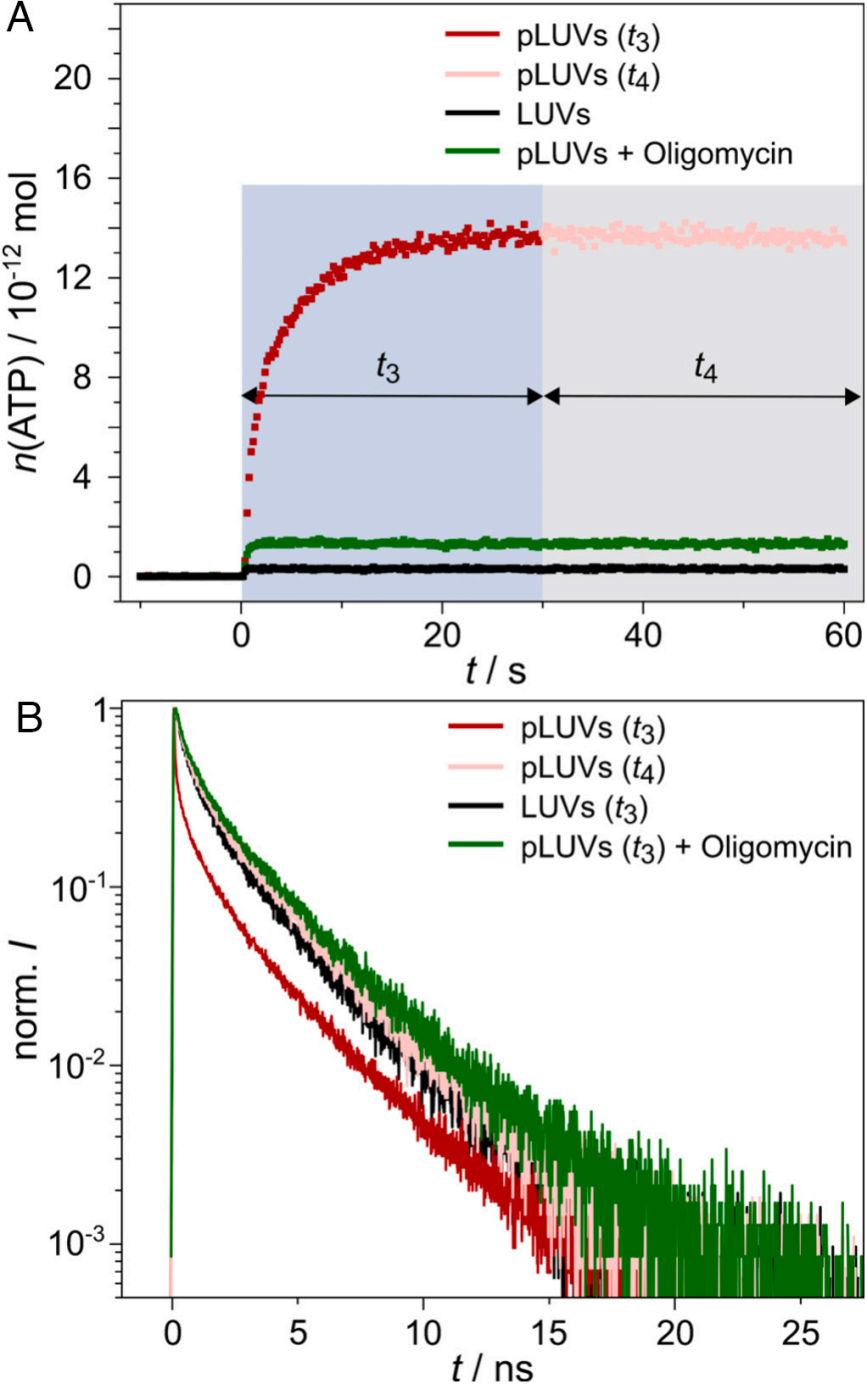
ATP synthesis activity in the absence and presence of the inhibitor oligomycin. (*A*) Luminescence spectroscopic measurements of ATP production as a function of time *t* for pLUVs with active TF_O_F_1_ ATP synthase (red squares) during *t*_3_ (blue box) and with inactive TF_O_F_1_ ATP synthase (light red squares) in phase *t*_4_ (gray box), for LUVs lacking the protein (black squares) and for pLUVs in the presence of oligomycin (green squares). (*B*) Time-resolved fluorescence of pLUVs during *t*_3_ (red) and *t*_4_ (light red), LUVs (*t*_3_, black) and pLUVs in the presence of oligomycin (*t*_3_, green).

To further substantiate that the observed ESPT changes of C_12_-HPTS are linked to TF_O_F_1_-ATP synthase activity, we monitored ESPT parameters after inhibiting the enzyme. We chose the commercially available inhibitor oligomycin, which interacts with the F_O_ subunit of ATP synthases (43). In contrast to other commercially available inhibitors such as N,N′-dicyclohexylcarbodiimide, oligomycin does not modify the charges of central glutamate residues in the F_O_ subunit (44). In general, oligomycin is a well-known inhibitor of mitochondrial ATP synthases (45). However, it has also been reported that it can inhibit bacterial ATP synthases such as those of *Rhodobacter capsulatus* (46) and the thermophilic *Bacillus* PS3 TF_O_F_1_-ATP synthase (47). To prove the inhibitory effect of oligomycin in our system, we first performed the luminescence assay of ATP production (Fig. 4*A*) in the presence of 67 μM oligomycin. Compared to the report by Weber et al. (47), we used a 2.7 times larger inhibitor concentration, as they did not use a reconstituted system but instead used inverted membrane vesicles. We measured a TON of 8.7 s^−1^ before oligomycin inhibition, which was reduced to 1.9 s^−1^ in the presence of the inhibitor. For comparison, Weber and coworkers reported a TON of 2.7 s^−1^ in their system and reported <0.1 s^−1^ in the oligomycin-inhibited case.

Second, we measured the time-resolved decay of C_12_-HPTS upon establishing the pmf in the presence of the oligomycin inhibitor (Fig. 4*B*). We obtained almost the same fluorescence decay as that measured for POPC LUVs lacking the ATP synthase and pLUVs at *t*_4_, where the ATP synthase does not produce ATP anymore (>1 min after pmf establishment). As a control, we measured the fluorescence decay in control POPC LUVs in the presence of oligomycin (*SI Appendix*, Fig. S7). The inhibitor does not influence the C_12_-HPTS decay. This experiment further supports our notion that the observed changes in the ESPT dynamics are directly correlated with the TF_O_F_1_-ATP synthase activity.

### Comparison of PT of C_12_-HPTS and Water-Soluble HPTS.

**Fig. 5.**
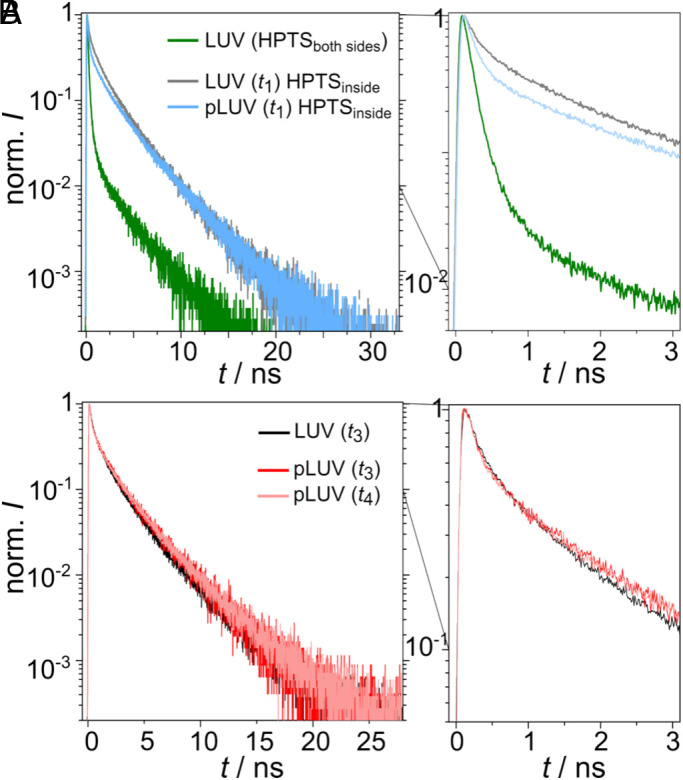
Time-resolved fluorescence decay of solvated HPTS. (*A*) LUVs with HPTS on both sides (green) and LUVs (gray) and pLUVs (light blue) with HPTS entrapped in the vesicles at pH 4.7 in phase *t*_1_. (*B*) Time-resolved fluorescence decay of entrapped HPTS in pLUVs directly after the pH switch (red, *t*_3_) and 2 min after the pH switch (light red, *t*_4_). LUVs without protein (black) in *t*_3_ are shown for comparison.

One of the significant advantages of the C_12_-HPTS probe is its ability to release a proton directly on the membrane surface, as it is membrane-tethered and interacts with its −OH group with moieties of the lipid headgroups (29). A few decades ago, a series of studies followed the PT of water-soluble HPTS (pyranine) from water to the surface of the membrane (48–51). In these studies, HPTS was solvated in the water phase of hydrated membranes, and the PT and proton recombination, presumably to/from the membrane, were observed to determine the “proton collecting antenna effect” of the membrane. In line with these studies, we aimed to compare the PT from solvated HPTS adjacent to the membrane with the PT we have found for C_12_-HPTS. When solvated HPTS is located on both sides of the LUVs, its PT to water is the dominant process and completely masks any membrane-interfacial PT in time-resolved measurements (Fig. 5*A*, green curve). Therefore, we confined the solvated HPTS to the inner lumen of the LUVs to observe PT events from HPTS to the membrane (structures C, Fig. 1). Interestingly, upon confining the solvated HPTS in the LUVs, the fluorescence decay changes considerably (Fig. 5*A*, grey curve). The fluorescence of the LUV-entrapped, solvated HPTS decays more slowly than that of free, solvated HPTS. We extracted the *k*_PT_-values and found indeed a decrease of *k*_PT_ from 9 ns^−1^ (29, 39) to 4.9 ns^−1^. This result indicates an interaction of the LUV-entrapped solvated HPTS with the membrane.

We then compared the results achieved for POPC LUVs with those obtained for POPC LUVs with reconstituted ATP synthase, both with entrapped HPTS. To ensure that the entrapment of HPTS into the vesicle lumen does not alter the parameters of the protein reconstitution, we determined the reconstitution efficiency (*R*_eff_ *=* 78 ± 12%, *N* = 9), the protein-to-lipid ratio (*p*/*l* = 1:21,000 ± 7,000, *N* = 9), the LUV diameter (2 *r*_pLUV_ = 139 ± 21, *N* = 9), the number of proteins per LUV (*N*_protein_ = 9 ± 3, *N* = 9), and the protein orientation (χ = 78%) (structures C.II, Table 1). All parameters were the same as those obtained for structures A.II and B.II. The TON = 8.0 ± 2.0 s^−1^ (C.II, *N* = 5) was also determined, showing that the activity of the TF_O_F_1_ ATP synthase is not influenced by the presence of solvated HPTS in the vesicle lumen. In addition, we determined the p*K*_a_ values of entrapped HPTS in both LUVs and pLUVs to be 7.1 and 7.2 for the ground state and 1.2 and 1.3 for the excited state (*SI Appendix*, Fig. S8 and Table S2), ensuring similar conditions to those of C_12_-HPTS.

The fluorescence decays obtained for solvated HPTS entrapped in pure POPC LUVs and in pLUV with reconstituted ATP synthase were only slightly different (Fig. 5*A*, grey and blue curves), which might be due to slightly different membrane electrostatics between the two membrane configurations. Upon activation of the ATP synthase by the pH switch (*t*_3_), we did not observe a remarkable change in the HPTS fluorescence decay (Fig. 5*B*, red curve). The decay is the same as that obtained for POPC LUVs without the enzyme (Fig. 5*B*, black curve) and for POPC LUVs with the enzyme, but at a time point where the enzyme is again inactive (Fig. 5*B*, light red curve).

## Discussion

Given the significance of PT at biological membranes, the mechanism by which protons are transported from a proton source to a proton sink has been a subject of research for decades (9). While in the early days, it was assumed that the protons that move across the membrane rapidly equilibrate with the bulk phase (52), in recent years, a picture has emerged showing that protons do not significantly equilibrate with the bulk but instead diffuse along the membrane surface to a proton acceptor (9, 10, 19, 21, 53). This model was based on the surprising finding that protons can move over micrometer distances along membranes. Protonation rates for targets at a membrane were two orders of magnitude larger than in bulk, reaching 10^13^ M^−1^s^−1^ (7, 11, 54), and a significantly extended proton lifetime was found at membrane surfaces (8, 55), defining the distance it can diffuse.

This behavior greatly impacts the coupling of a proton release site and a proton consumer site as found in bioenergetic processes, where proton pumps and proton consumers, such as the ATP synthase, are intimately coupled. Indeed, we found that the activity of the TF_O_F_1_ ATP synthase serves as a perfect sink for protons released on the membrane surface. Protons dissociated from the photoacid undergo an unexpectedly fast PT when the ATP synthase consumes protons to produce ATP. The enzyme reduces the dimensionality of PD strictly to the membrane surface (*d* ≤ 2) and increases the PD coefficient. Hence, we conclude that the enzyme fundamentally changes the role of the membrane in PT and PD.

**Fig. 6.**
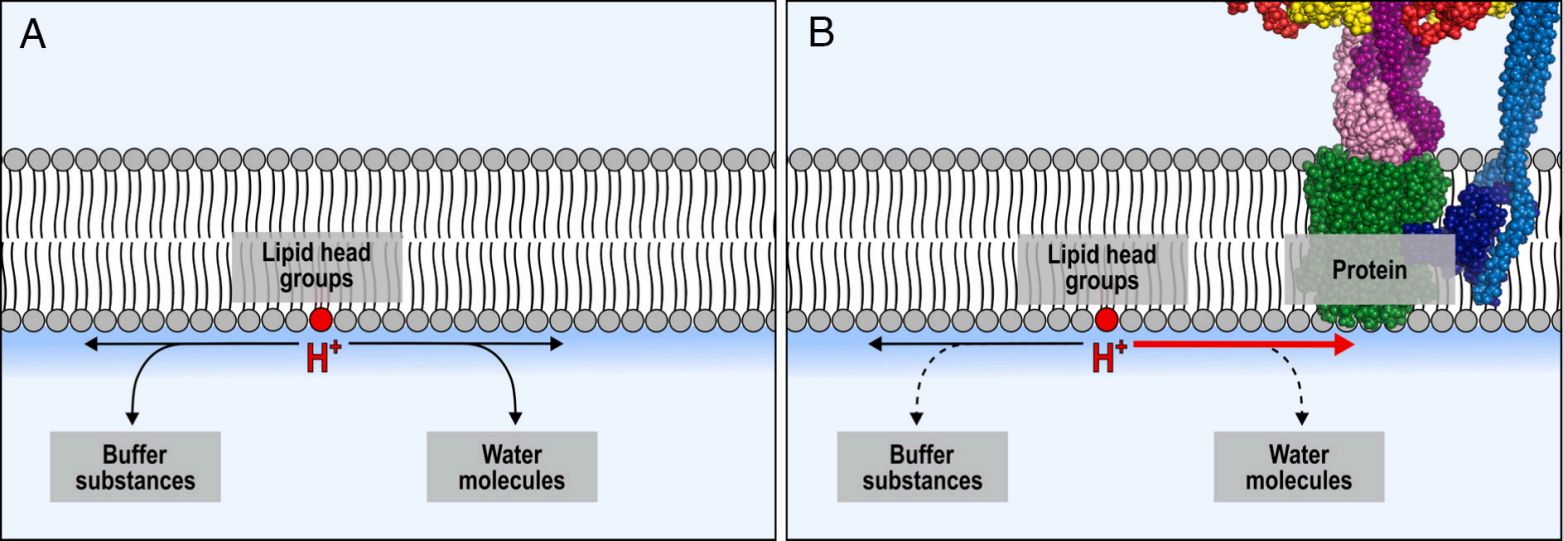
Comparison of the PT processes for C_12_-HPTS in the absence (LUVs, *A*) and the presence (pLUVs, *B*) of active ATP synthase. The shown processes are valid for the excited state lifetime of the photoacid (ns). (*A*) Protons are released at the membrane surface, where they remain temporarily attached. Occasionally, escape events occur, in which protons diffuse into the bulk medium, indicated by a diffusion dimensionality of 2 < *d* < 3. (*B*) Protons released at the membrane surface by C_12_-HPTS exhibit an extremely fast PT rate from the probe to the membrane, indicating the presence of an additional proton sink introduced by the active enzyme. Moreover, the PD dimensionality decreases to *d* ≤ 2, implying that membrane-to-bulk escape events no longer occur. Instead, protons remain associated with the membrane and diffuse towards the ATP synthase via the membrane interface without exchanging with bulk protons.

Our findings of an ultra-fast PT from the tethered probe to the surface of the membrane and the consequent change in the PD dimensionality only upon the activation of the ATP synthase provide evidence that the proton consumption of the active ATP synthase generates a purely 2D proton gradient along which the protons diffuse toward the enzyme (Fig. 6), i.e., they do not exchange with the bulk. It is important to reiterate that this conclusion is valid for the ESPT processes from the tethered photoacid that is used here as a local probe to explore the properties of the membrane as a function of the ATP synthase activity, while the dissociated protons from the photoacid do not contribute to the established pmf for the initiation of the enzyme activity. While referring to the mentioned quasi-equilibrium vs. non-equilibrium models for PD on the surface of the membrane, our findings might suggest that upon activating the ATP synthase, there is a shift from the quasi- to the non-equilibrium behavior of the proton. However, to date, there is no experimental system in literature that can specifically resolve this conflict. The observed PT behavior exceeds previously reported findings. Aforementioned studies have explored lateral diffusion along membrane surfaces, including lipid monolayers (56), lipid bilayers (57), and small unilamellar vesicles (26). In these analyses, the proton lateral diffusion approached that of free protons in bulk water. In a study by Pohl et al., the PD coefficient on the membrane even matched or exceeded that in bulk water (13). At present, we are not fully able to describe the diffusive behavior when a proton consumer is incorporated into the membrane system, and further studies may be required.

## Materials and Methods

### Materials.

POPC was purchased from Avanti Research (Alabaster, USA). Dodecyl-β-d-maltoside (DDM) and *n*-octyl-β-d-glucopyranoside (*n*-OG) were obtained from Carl Roth (Karlsruhe, Germany), heptakis(2,6-di-O-methyl)-β-cyclodextrin (mβCD) from Fisher Scientific GmbH (Schwerte, Germany). Bio-Beads SM2 were purchased from Bio-Rad Laboratories GmbH (Feldkirchen, Germany) Sephadex G-25 prepacked illustra NAP-10 columns from GE HealthCare Technologies (Chicago, USA). C_12_-HPTS was synthesised as reported previously (29).

### Isolation and Expression of the TF_O_F_1_-ATP Synthase.

TF_O_F_1_-ATP synthase was expressed in *Escherichia* *coli* DK8 containing the plasmid pTR19-ASDS, encoding for the His-tagged ATP synthase of the thermophilic *Bacillus* strain PS3 (58). Expression and purification were performed as described previously (59) (*SI Appendix*, Fig. S9 *A–C*). The purity of the protein samples was analysed using SDS-PAGE (*SI Appendix*, Fig. S9*D*).

### Preparation of POPC-LUV (Structures I).

Structure A.I: POPC was transferred from a chloroform stock solution into a small test tube. Chloroform was evaporated under a nitrogen stream, and the resulting lipid film was dried in vacuo for at least 3 h at room temperature. To prepare multilamellar vesicles (MLVs) the lipid film (5 mg POPC) was incubated with vesicle buffer (0.5 mL, 20 mM Tricin, 20 mM succinic acid, 2.5 mM MgCl_2_, 0.6 mM KCl, pH 8.0) for 30 min and subsequently vortexed three times for 30 s in intervals of 5 min. LUVs were prepared from MLVs via extrusion through a polycarbonate membrane with a nominal diameter of 100 nm. Structure B.I: For POPC/C_12_-HPTS (99/1) mixtures, the desired mol fraction of C_12_-HPTS was added from an ethanol stock solution during lipid film preparation. LUVs were prepared from the lipid film as for structure A.I. Structure C.I: A POPC film (5 mg) was rehydrated in vesicle buffer containing water-soluble HPTS (2 mM). After preparation of the LUVs as described for structure A.I., HPTS was removed from the outside solution by gel filtration (Sephadex G-25 illustra NAP-10 column).

### TFOF1-ATP Synthase Reconstitution into POPC-LUVs (Structures II).

LUVs were first prepared as described above for structures A.I-C.I. Then, *n*-OG with a critical micelle concentration (cmc) of 25 mM (60) was added to the LUVs and incubated on a rotary mixer for 30 min at room temperature. The *n*-OG concentration (*c_n_*_-OG_) was chosen so that *R* = (*c*_n-OG_ – cmc)/*c*_lipid_, with *c*_lipid_, the lipid concentration, equals one, ensuring a LUV destabilisation that leads to a unidirectional reconstitution of the TF_O_F_1_-ATP synthase (37, 59). The ATP synthase was added at a nominal protein-to-lipid ratio of 1:20,000 and further incubated on a rotary mixer for 30 min at room temperature, followed by dialysis overnight at 4 °C against 1 L vesicle buffer supplemented with Bio-Beads SM2. To remove residual DDM from the protein purification, an equimolar amount of mβCD was added and incubated for 1 h on ice. The mβCD-complexed DDM was removed by gel filtration through a Sephadex G-25 prepacked illustra NAP-10 column (this step also removed the water-soluble HPTS, compare structures C.I and C.II). Phospholipid concentrations of the proteo-LUVs (pLUVs) were determined by a phosphate test (59, 61). The reconstitution efficiency of the TF_O_F_1_-ATP synthase-containing vesicles was determined by density gradient centrifugation (*SI Appendix*, Fig. S1), and the average size of the pLUVs by dynamic light scattering measurements (Zetasizer Nano S, Malvern Panalytical, Worcestershire, United Kingdom). Protein orientation was investigated by digestion experiments using the protease Proteinase K (*SI Appendix*, Fig. S2). The integrity of the protein was investigated by blue native PAGE (*SI Appendix*, Fig. S10).

### Luciferase-Luciferin Assay.

ATP production was measured using a luciferin-luciferase assay from the American firefly *Photinus pyralis* (ATP Bioluminescence Assay Kit CLS II, Roche, Basel, Switzerland) via luminescence spectroscopy (Tristar 5, Berthold Technologies, Bad Wildbad, Germany). LUVs and pLUVs were acidified for 10 min in LI-buffer (20 mM succinic acid, 5 mM NaH_2_PO_4_, 2.5 mM MgCl_2_, 0.6 mM KOH, pH 4.7) in the presence of valinomycin [valinomycin-to-lipid ratio (*val*/*l*) of 1:40] and ultra-pure ADP (350 µM). Upon addition of LII buffer (200 mM Tricine, 160 mM KOH, 5 mM NaH_2_PO_4_, 2.5 mM MgCl_2_, pH 8.8), a pH gradient of ΔpH = 4.1 and a membrane potential facilitated by K^+^ of Δ ψ = 128 mV was applied resulting in a total pmf of 373 mV, which drives ATP production through the TF_O_F_1_-ATP synthase. In the presence of ATP, luciferase catalyzes the conversion of D-luciferin to excited-state oxyluciferin, which emits light with a wavelength of 562 nm as it returns to its ground state (62, 63). For the inhibition experiments, LUVs and pLUVs were acidified for 60 min in LI-buffer supplemented with an additional 200 µM oligomycin. Subsequently, LII buffer was added at twice the volume of the acidic bath resulting in a final oligomycin concentration of 67 µM. All measurements were performed at *T* = 27.5 °C. For quantification, an internal calibration was performed by adding ATP standards.

### Fluorescence Spectroscopic Measurements.

LUVs and pLUVs were acidified for 10 min in LI-buffer in the presence of valinomycin (*val*/*l* of 1:40) and ultra-pure ADP (350 µM). For the inhibition experiments, LUVs and pLUVs were acidified for 60 min in LI-buffer supplemented with an additional 200 µM oligomycin. Subsequently, LIIa buffer (200 mM Tricine, 160 mM KOH, 5 mM NaH_2_PO_4_, 2.5 mM MgCl_2_, pH 7.4) was added at twice the volume of the acidic bath. Steady-state fluorescence of both LUVs and pLUVs was performed using an FS5 spectrofluorometer (Edinburgh Instrument). Time-correlated single photon counting (TCSPC) experiments were performed using a 1,030 nm and 10 W Yb-based PHAROS laser (Light Conversion) with a pulse duration of 190 fs. The laser beam was seeded into an optical parametric amplifier (ORPHEUS), after which it was seeded into a spectrometer (CHIMERA), where the time-resolved spectrum was measured in a single photon counting technique using a hybrid detector (Becker & Hickl, HPM-100-07) that has an instrument response function (IRF) of ~50 ps at full-width-half-maximum. The excitation wavelength for both steady-state and TCSPC measurements was 400 nm. The emission wavelengths for the TCSPC measurements were 470 and 550 nm for ROH^*^ and RO^−*^, respectively. All measurements were performed at room temperature (24 °C).

## Supplementary Material

Appendix 01 (PDF)

## Data Availability

All other data are included in the manuscript and/or *SI Appendix*.
