# Supplementary material for: ATP synthase activity boosts membrane proton acceptance and lateral diffusion
Source: Proc Natl Acad Sci U S A. 2026 Mar 3;123(10):e2510444123. doi: 10.1073/pnas.2510444123 (PMC12974407; doi:10.1073/pnas.2510444123)
Supplement: Supplementary file 1 — Appendix 01 (PDF) [file pnas.2510444123.sapp.pdf]

PNAS

Supporting Information for

ATP Synthase Activity Boosts Membrane Proton Acceptance and Lateral Diffusion

Hendrik Flegel,[1] Ambili Ramanthrikkovil Variyam,[2] Nadav Amdursky,[2,3,*] Claudia Steinem[1,*]

[1]Institute of Organic and Biomolecular Chemistry, University of Göttingen, Tammannstr. 6, 37077 Göttingen, Germany

[2]Schulich Faculty of Chemistry, Technion – Israel Institute of Technology, Haifa 3200003, Israel

[3]School of Mathematical and Physical Sciences, University of Sheffield, 13 Brook Hill, Sheffield S3 7HF, United Kingdom

* Nadav Amdursky, Claudia Steinem

Email: n.amdursky@sheffield.ac.uk; csteine@gwdg.de

This PDF file includes:

Supporting text
Figures S1 to S10
Table S1 to S2
SI References

Supporting Information Text

Determination of the reconstitution efficiency by density gradient centrifugation

The total amount of reconstituted protein, i.e., the reconstitution efficiency, was determined by performing density gradient centrifugations of the pLUVs in a Histodenz gradient (0-40% (*w*/*v*)) in vesicle buffer. To prepare the gradient, 40 µL of an 80% (*w*/*v*) Histodenz solution was mixed with 40 µL of pLUVs, followed by an overlay of 40 µL of a 30% (*w*/*v*) Histodenz solution, and finally, 20 µL of vesicle buffer. This gradient was centrifuged at 50,000 × *g* for 1.5 hours at 4°C. Intact vesicles containing vesicle buffer were expected to float to the top due to their lower density, and thus, were found in the upper fractions (F1-4). Non-inserted protein was found in the lower fractions (F5-7). The reconstitution efficiency R_{eff} was assessed by comparing the band intensities of the α- and β-subunits (Fig. S1).

Additionally, three free protein standards (PS1: 0.5 pmol, PS2: 1.5 pmol, PS3: 2.5 pmol) were used to determine the absolute amount of inserted TF_OF_1 ATP synthase $c_{protein}$. The intensities of the protein bands from standards PS1-3 served as a calibration to quantify the amount of protein successfully reconstituted in the vesicle membranes (Fig. S1).

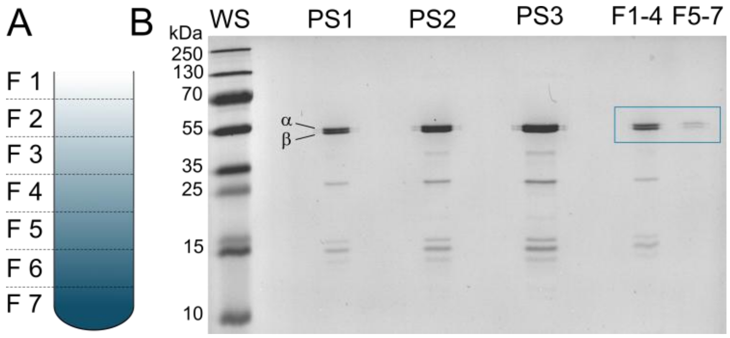

Fig. S1. Determination of the TF_OF_1 ATP synthase reconstitution efficiency. (*A*) The Histodenz gradient (0-40 % (*w*/*v*)) was prepared in vesicle buffer (20 mM Tricin, 20 mM succinic acid, 2.5 mM $MgCl_2$, 0.6 mM KCl, pH 8.0). (*B*) The SDS-PAGE gel from the reconstitution of pLUVs with a nominal protein-to-lipid ratio of p/l 1:20,000 contains a prestained weight standard (WS), three protein standards (PS1: 0.5 pmol, PS2: 1.5 pmol, PS3: 2.5 pmol TF_OF_1 ATP synthase), and combined samples of the density gradient fractions F1-4 and F5-7. For quantification, band intensities of the α- and β-subunit were used for calibration. Comparison of F1-4 and F5-7 band intensities allowed for calculating the reconstitution efficiency (blue box).

Vesicle size determination

Table S1. Comparison of the vesicle diameters $2r_{LUVs}$ obtained for LUVs composed of POPC (A.I), POPC/C_{12}-HPTS (B.I), and soluble HPTS encapsulated in LUVs (C.I) (mean ± standard deviation).

Structure	$2r_{LUVs}$ / nm
**A.I**	130 ± 31
**B.I**	130 ± 30
**C.I**	132 ± 17

Calculation of the ATP synthase molecules per vesicle

To estimate the average number of TF_OF_1 ATP synthase molecules per vesicle ($N_{protein}$), several parameters are required: the mean diameter of the pLUVs ($2r_{pLUVs(A.II)}$ = 145 nm, $2r_{pLUVs(B.II)}$ = 137 nm) and the protein-to-lipid ratios ($p/l_{pLUVs(A.II)}$ = 1:27,000, $p/l_{pLUVs(B.II)}$ = 1:25,000). The area of a POPC lipid is assumed to be a_{POPC} = 0.63 nm² (1) and that of an ATP synthase $a_{Protein}$ = 20 nm² (2). The area fraction of the protein $f_{Protein}$ of the total surface area of the vesicle membrane was calculated using Eq. S1:

$$f_{\mathrm{Protein}} = \frac{a_{\mathrm{Protein}}}{a_{\mathrm{Protein}} + p/l_{\mathrm{pLUVs}} \cdot a_{\mathrm{POPC}}}. \tag{S1}$$

Using $f_{Protein}$, the total membrane surface area occupied by the ATP synthase $A_{Protein}$ can be calculated using Eq. S2:

$$A_{\mathrm{Protein}} = f_{\mathrm{Protein}} \cdot (A_{\mathrm{outer\ leaflet}} + A_{\mathrm{inner\ leaflet}}). \tag{S2}$$

The area of the outer membrane leaflet is $A_{\mathrm{outer\ leaflet}} = \pi \cdot (2r_{pLUVs})^2$. The area of the inner leaflet can be calculated as $A_{\mathrm{inner\ leaflet}} = \pi \cdot (2r_{pLUVs} - 2h_{\mathrm{Bilayer}})^2$, with h_{Bilayer} = 3.98 nm (1) being the thickness of the POPC bilayer. The number of lipids per vesicle N_{Lipid}, and the number of proteins per vesicle N_{Protein} can be derived using Eq. S3:

$$N_{\mathrm{Protein}} = \frac{N_{\mathrm{Lipid}}}{p/l_{\mathrm{pLUVs}}}, \tag{S3}$$

with $$N_{\mathrm{Lipid}} = \frac{(A_{\mathrm{outer\ leaflet}} + A_{\mathrm{inner\ leaflet}} - A_{\mathrm{Protein}})}{a_{\mathrm{POPC}}}.$$

Determination of the protein orientation of reconstituted TF_OF_1 ATP synthase

Because proton translocation through the ATP synthase is unidirectional, the protein's orientation is of utmost importance. We assessed orientation by proteinase K digestion. When the F_1 subunit of the reconstituted ATP synthase faces the extravesicular medium, the α- and β-subunits are accessible to proteinase K and susceptible to proteolysis. If the F_1 subunit faces the LUV lumen, the membrane shields this subunit from the protease. The fraction of α- and β-subunits digested thus reports on the protein orientation and was quantified by SDS-PAGE and Western blot analysis (Fig. S2). Band intensities for the α- and β-subunits were compared across three conditions: pLUVs without proteinase K treatment ($I_{(a)}$), pLUVs treated with proteinase K (0.01 mg/mL) for 15 h at 25 °C ($I_{(b)}$), and ATP synthase in the absence of LUVs treated with proteinase K (0.01 mg/mL) for 15 h at 25 °C ($I_{(c)}$). Following protease treatment, 5 µL of the protease inhibitor phenylmethylsulfonyl fluoride (PMSF, 12 mg/mL) was added and incubated for 15 min at room temperature to quench digestion. All three samples were analyzed by SDS-PAGE (Fig. S2*A*) and Western blot (Fig. S2*B*). The intensities of the α- and β-subunits from the TF_OF_1 ATP synthase extracted from the SDS-PAGE and Western blot were used to calculate the protein orientation χ (Eq. S4):

$$\chi = 1 - \frac{I_{(b)} - I_{(c)}}{(I_{(a)} - I_{(c)}) \cdot R_{\mathrm{eff}}}. \tag{S4}$$

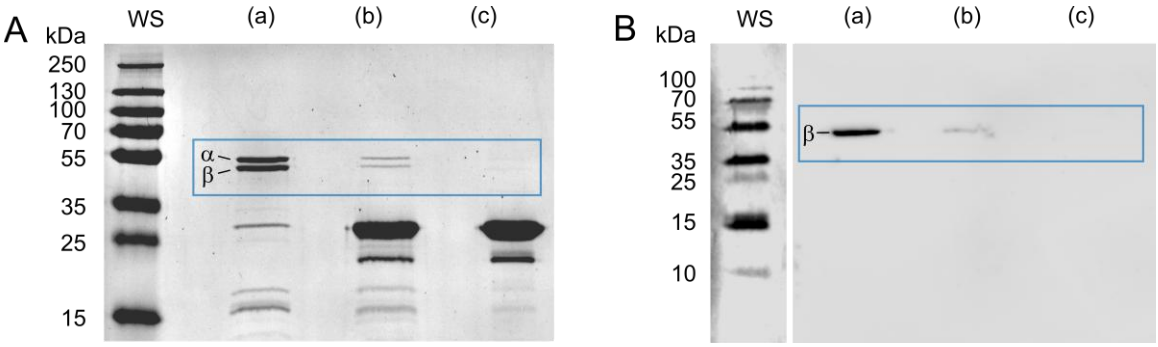

Fig. S2. Determination of the ATP synthase orientation in pLUVs. (*A*) SDS-PAGE gel with prestained weight standard (WS) and (*B*) Western blot of the TF_OF_1 ATP synthase reconstituted in POPC LUVs (a) without proteinase K, (b) after treatment with proteinase K for 15 h, and (c) TF_OF_1 ATP-synthase in the absence of LUVs after treatment with proteinase K for 15 h. The Western blot was examined using the monoclonal mouse antibody against the ATP synthase subunit β (0.5 µg/ml) and detected by chemiluminescence using a polyclonal secondary goat anti-mouse IgG antibody, a horseradish peroxidase (HRP) conjugate (0.25 µg/ml), and an ECL Prime Western blotting detection reagent. The WS was prestained.

Determination of pK_a of C_{12}-HPTS in ground- and excited-state

To determine the ground-state pK_a value of the photoacids C_{12}-HPTS (Fig. S3) and solvated HPTS (Fig. S8), absorbance spectra at different pH values were recorded for LUVs (*A1*) and pLUVs (*A2*). For this purpose, the data was normalized to $I_{\mathrm{Abs}}^{\mathrm{min}} = 0$ and $I_{\mathrm{Abs}}^{\mathrm{max}} = 1$ for ROH and *vice versa* for RO^-. Relative absorbance intensities at the marked peaks for ROH and RO^- were plotted against the respective pH value (*B1*: LUVs, *B2*: pLUVs). Eq. S5 was used as a fitting function to calculate the ground-state pK_a:

$$\mathrm{rel.}\ I_{\mathrm{Abs}} = I_{\mathrm{Abs}}^{\mathrm{min}} + \frac{I_{\mathrm{Abs}}^{\mathrm{max}} - I_{\mathrm{Abs}}^{\mathrm{min}}}{1 + 10^{\mathrm{p}K_{\mathrm{a}} - \mathrm{pH}}} \tag{S5}$$

Emission spectra of the photoacids C_{12}-HPTS (Fig. S3*C*) and soluble HPTS (Fig. S8*C*) at different pH values were utilized to calculate the change in acidity after excitation. Therefore, the peak maxima of both protonated and deprotonated species in absorbance spectra $\left(\lambda_{\mathrm{Abs}}^{\mathrm{ROH}}\right.$ and $\left.\lambda_{\mathrm{Abs}}^{\mathrm{RO^-}}\right)$ and emission spectra $\left(\lambda_{\mathrm{Em}}^{\mathrm{ROH}}\right.$ and $\left.\lambda_{\mathrm{Em}}^{\mathrm{RO^-}}\right)$ were expressed in terms of vertical transition energies *E* using Eq. S6, with *h* and c_0 denoted as usual:

$$E = \frac{hc_0}{\lambda} \tag{S6}$$

The energy gaps ΔE of the vertical electronic transitions were determined using Eq. S7:

$$\Delta E^{\mathrm{ROH}} = \frac{E_{\mathrm{Abs}}^{\mathrm{ROH}} + E_{\mathrm{Em}}^{\mathrm{ROH}}}{2}, \qquad \Delta E^{\mathrm{RO^-}} = \frac{E_{\mathrm{Abs}}^{\mathrm{RO^-}} + E_{\mathrm{Em}}^{\mathrm{RO^-}}}{2} \tag{S7}$$

R, *T,* and N_A, the Avogadro constant, are denoted as usual.

The change in acidity $\Delta \mathrm{p}K_{\mathrm{a}}$, and the excited-state $\mathrm{p}K_{\mathrm{a}}^*$ were estimated with Eqs. S8 and S9:

$$\Delta \mathrm{p}K_{\mathrm{a}} = \frac{\left(\Delta E^{\mathrm{ROH}} - \Delta E^{\mathrm{RO^-}}\right) N_{\mathrm{A}}}{\ln(10) RT} \tag{S8}$$

$$\mathrm{p}K_{\mathrm{a}}^* = \mathrm{p}K_{\mathrm{a}} - \Delta \mathrm{p}K_{\mathrm{a}} \tag{S9}$$

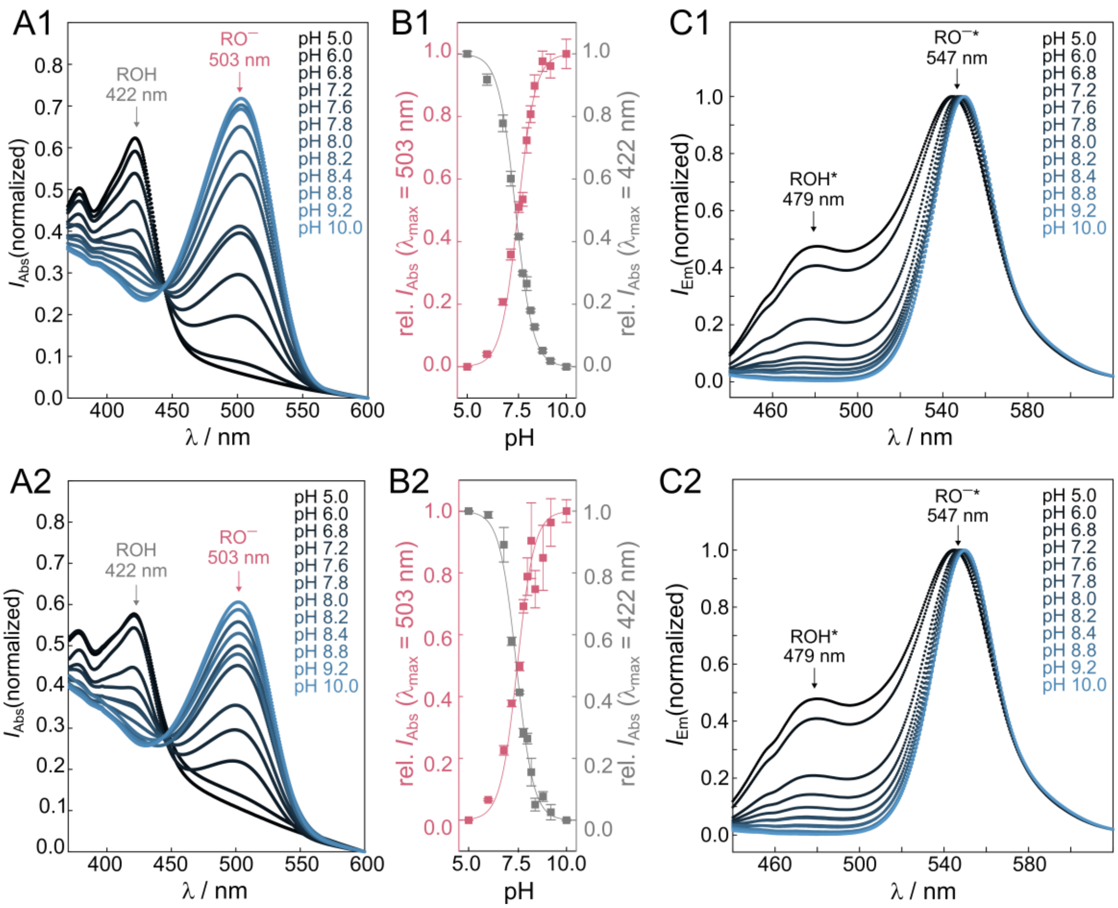

Fig. S3. Spectroscopic data of C_{12}-HPTS embedded in the vesicle membrane. (*A*) Absorbance spectra of C_{12}-HPTS incorporated in the lipid membranes of POPC LUVs (*A1*) and TF_OF_1 ATP synthase-containing pLUVs (*A2*) for different pH values between pH 5.0 (black) and pH 10.0 (blue) for $N \geq 1$ vesicle preparations, i.e., protein reconstitutions. Absorption maxima were found at λ_{max}^{ROH} = 422 nm for the protonated species and $\lambda_{max}^{RO^-}$ = 503 nm for the deprotonated species in LUVs and pLUVs. T = 20 °C. (*B*) Relative absorbance intensity I_{Abs} at λ_{max}^{ROH} = 422 nm (gray squares) and $\lambda_{max}^{RO^-}$ = 503 nm (magenta squares) of C_{12}-HPTS incorporated in lipid membranes of POPC LUVs (*B1*) and TF_OF_1 ATP synthase-containing pLUVs (*B2*) as a function of pH. Eq. S5 was fitted to the data to extract the ground-state pK_a. T = 20 °C. The error bars are the standard deviation of the different preparations. (*C*) Fluorescence spectra of C_{12}-HPTS incorporated in lipid membranes of POPC LUVs (*C1*) and TF_OF_1 ATP synthase-containing pLUVs (*C2*) for different pH values between pH 5.0 (black) and pH 10.0 (blue) for $N \geq 1$ vesicle preparations, i.e., protein reconstitutions. Emission maxima were found at $\lambda_{max}^{RO^*}$ = 479 nm for the protonated species and $\lambda_{max}^{RO^{-*}}$ = 547 nm for the deprotonated species in LUVs and pLUVs. λ_{ex} = 395 nm, T = 20 °C.

Table S2. Summary of the pK_a values of C_{12}-HPTS and vesicle-entrapped soluble HPTS in LUVs and pLUVs.

	**pK_a**	**ΔpK_a**	**pK_a^***
**C_{12}-HPTS in LUVs**	7.5 ± 0.1	6.8	0.7
**C_{12}-HPTS in pLUVs**	7.5 ± 0.1	6.8	0.7
**HPTS in LUVs**	7.1 ± 0.1	5.9	1.2
**HPTS in pLUVs**	7.2 ± 0.3	5.9	1.3

Influence of excited-state proton release on *pmf*

The luciferase-luciferin assay was used to determine if light-induced geminate proton release contributes to the proton motive force (*pmf*) and subsequent ATP synthesis (Fig. S4). A comparison of ATP yields between light-exposed samples and those incubated in the dark displayed no significant difference. This result shows that protons released by 1 mol% C_{12}-HPTS did not affect ATP production, indicating that the *pmf* is not significantly altered. It is the established pH gradient that defines the *pmf*.

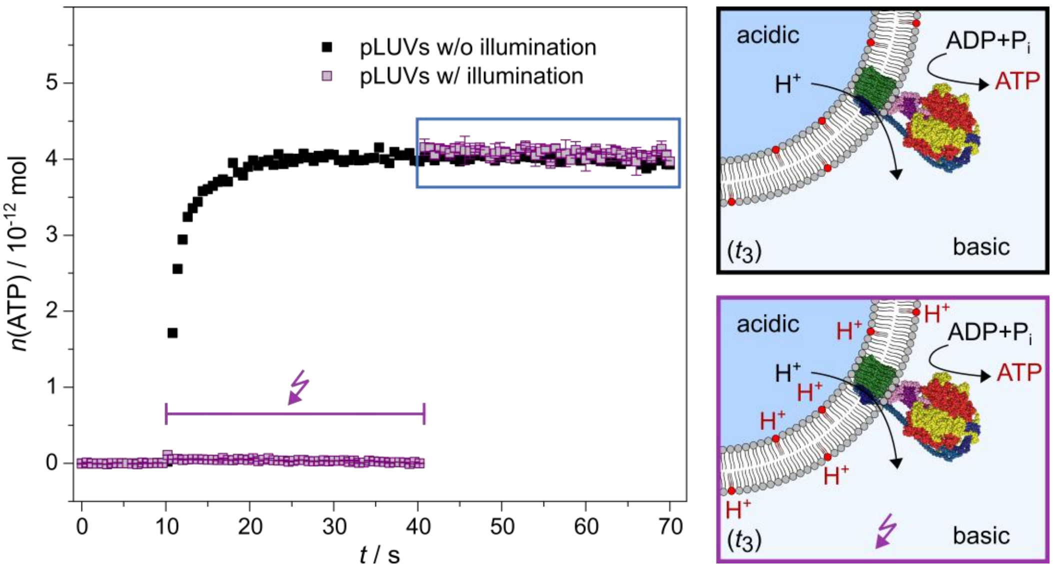

Fig. S4. Time trace of ATP production as a function of time *t* for light-exposed C_{12}-HPTS-containing (1 mol%) pLUVs and those incubated in the dark. pLUVs (structural motif B.II) were illuminated in the presence of the *ATP Bioluminescence Assay Kit CLS II* with an external nominal *pmf* of 373 mV (ΔpH = 4.1, $\Delta\Psi$ = 128 mV). The graphs display the luminescence intensities of pLUVs without illumination as a control (black squares) and with illumination (purple squares). Illumination takes place in the time window of 10-40 s with λ_{ex} = 422 nm, indicated by the purple flash. Comparison of the luminescence intensity after illumination (blue box) revealed that no increased ATP production caused by light-triggered proton release was recorded.

Results of the fitting routines for the TCSPC data

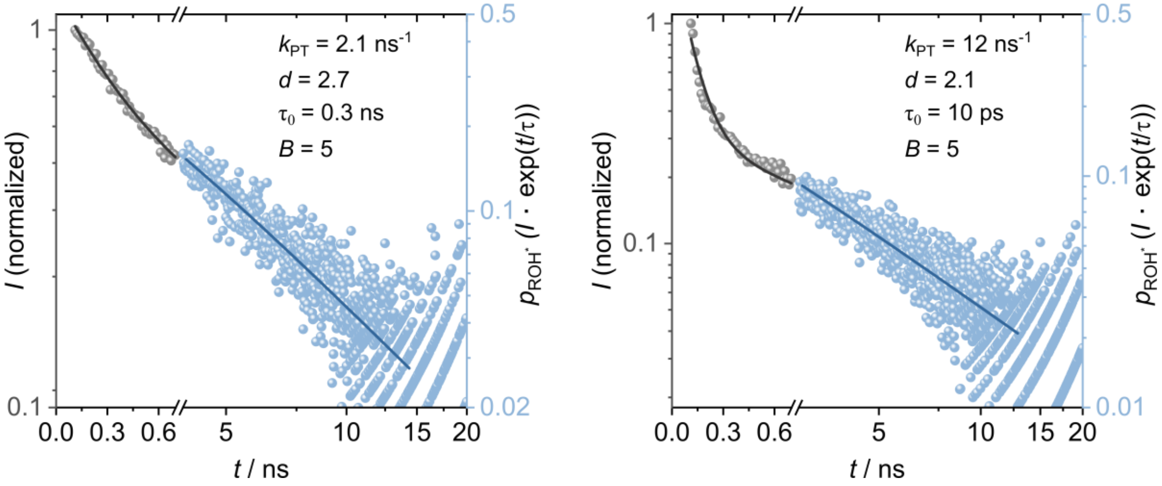

Fig. S5. Exemplary TCSPC data of the ROH* species of the C12-HPTS probe. The curves show the fluorescence decay of C12-HPTS reconstituted in LUVs (left) and pLUVs when the protein is active (right) (black and blue data points). The results of the fitting routines for the short time scale (black solid line) and the long time scale (blue solid line) are shown. The fitting was performed using the described model to extract k_{PT} and d, and τ_0 in both cases. λ_{ex} = 400 nm, λ_{em} = 470 nm.

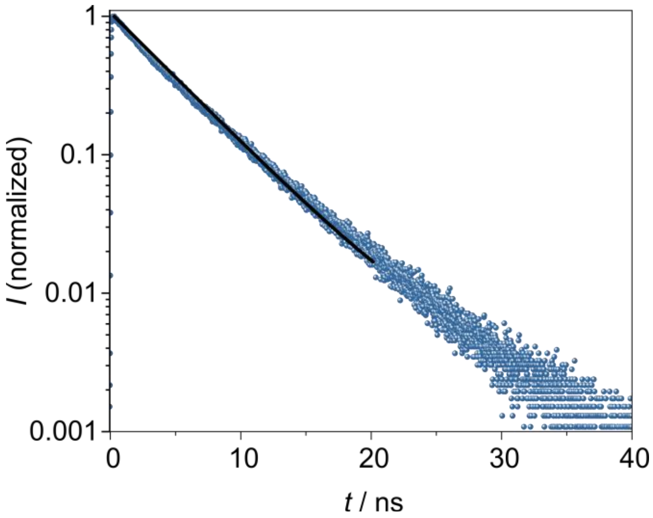

Fig. S6. Exemplary TCSPC data of the RO⁻* species of the C12-HPTS probe. Fitting a monoexponential decay curve allows the determination of k_{rx}. λ_{ex} = 400 nm, λ_{em} = 550 nm.

Influence of the TF_OF_1 ATP synthase inhibitor oligomycin on LUVs

Control experiment demonstrating that the presence of oligomycin, an inhibitor of ATP synthesis, does not affect the TCSPC of LUVs doped with 1 mol% C_{12}-HPTS.

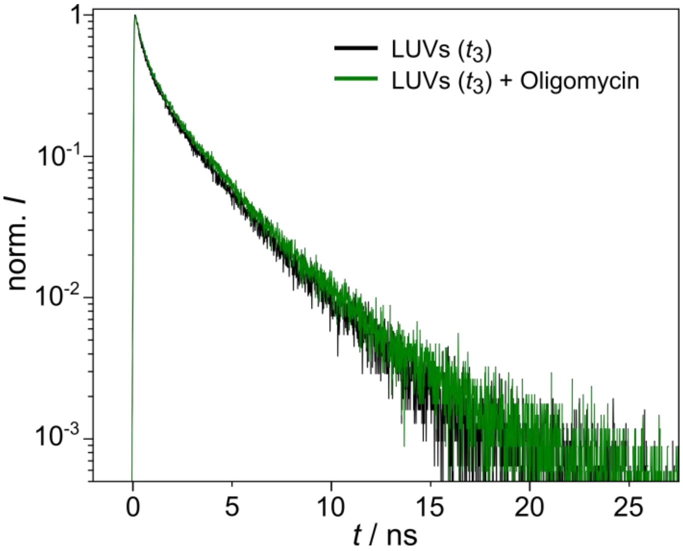

Fig. S7. Time-resolved fluorescence decay of C_{12}-HPTS in LUVs (structure B.I) during the phase t_3 in the absence (black) and presence (green) of 67 µM oligomycin.

Determination of pK_a of solvated HPTS in ground- and excited-state

The same procedure as described for the determination of the pK_a values of C_{12}-HPTS in the ground- and excited-state was applied.

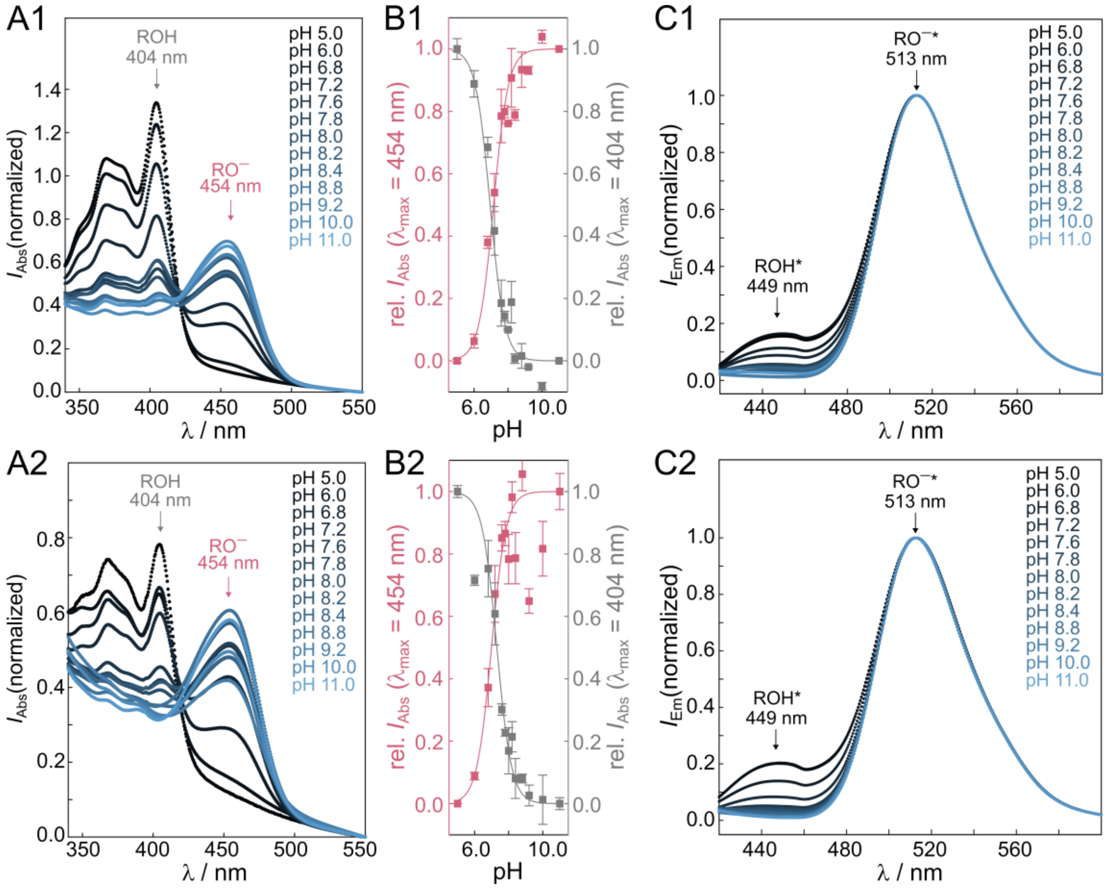

Fig. S8. Spectroscopic data of solvated HPTS entrapped in the vesicle lumen. (*A*) Absorbance spectra of HPTS entrapped in the lumen of POPC LUVs (*A1*) and TF_OF_1 ATP synthase-containing pLUVs (*A2*) for different pH values between pH 5.0 (black) and pH 11.0 (blue) for $N \geq 1$ vesicle preparations, i.e., protein reconstitutions. Absorption maxima were found at λ_{max}^{ROH} = 404 nm for the protonated species and $\lambda_{max}^{RO^-}$ = 454 nm for the deprotonated species in LUVs and PLUVs. T = 20 °C. (*B*) Relative absorbance intensity I_{Abs} at λ_{max}^{ROH} = 404 nm (gray squares) and $\lambda_{max}^{RO^-}$ = 454 nm (magenta squares) of HPTS entrapped in POPC LUVs (*B1*) and TF_OF_1 ATP synthase-containing pLUVs (*B2*) as a function of pH. Eq. S5 was fitted to the data sets to extract the ground-state pK_a. T = 20 °C. The error bars are the standard deviation of the different preparations. (*C*) Fluorescence spectra of HPTS entrapped in the lumen of POPC LUVs (*C1*) and TF_OF_1 ATP synthase-containing pLUVs (*C2*) for different pH values between pH 5.0 (black) and pH 11.0 (blue) for $N \geq 1$ number of vesicle preparations, i.e., protein reconstitutions. Emission maxima were found at $\lambda_{max}^{RO^*}$ = 449 nm for the protonated species and $\lambda_{max}^{RO^{-*}}$ = 513 nm for the deprotonated species in LUVs and pLUVs. λ_{ex} = 395 nm, T = 20 °C.

Expression and purification of the TF_OF_1 ATP synthase

Expression and purification of the TF_OF_1-ATP synthase (Fig. S9) was performed in *E. coli* DK8 harboring the plasmid pTR19-ASDS, encoding for the His-tagged ATP synthase of the thermophilic *Bacillus* strain PS3 as described previously (3).

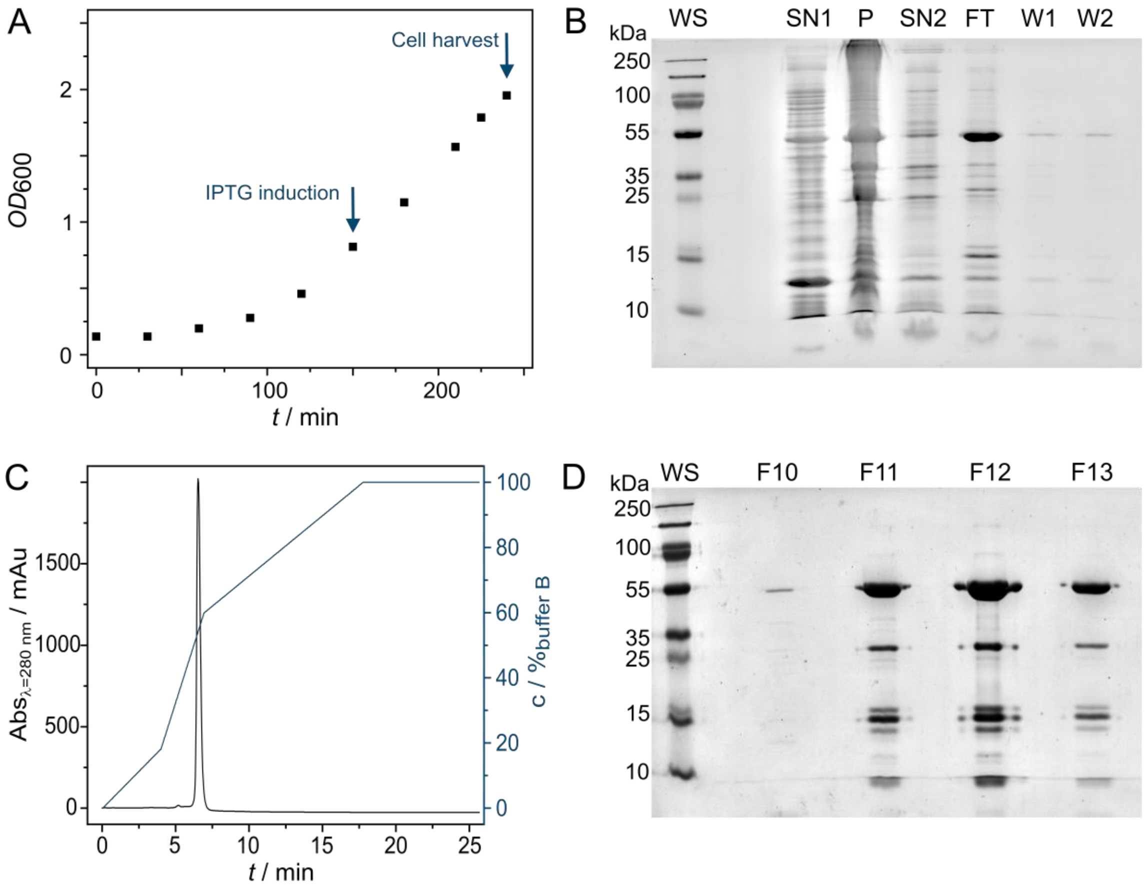

Fig. S9. Expression and purification of the TF_OF_1 ATP synthase. (*A*) Cell growth of the plasmid pTR19-ASDS containing *E. coli* DK8 cells monitored as an optical density at λ = 600 nm (OD_{600}) as a function of time. At OD_{600} = 0.8, IPTG was added to start protein production. Cells were harvested at OD_{600} = 2.0. (*B*) SDS-PAGE of samples obtained at different stages of protein isolation. WS: prestained weight standard, SN: supernatant, P: pellet, FT: flow through, W: wash fractions. (*C*) Elution profile (A_{280}, solid black line) of the TF_OF_1 ATP synthase during the elution procedure using anion exchange chromatography with increasing NaCl concentration (c% buffer B, blue solid line) (*D*) SDS-PAGE of the elution fractions of the anion exchange chromatography. WS: prestained weight standard, F: elution fractions. The respective bands can be assigned to the different TF_OF_1 subunits according to the literature (4).

Blue native PAGE (Fig. S10*A*) followed by 2D-SDS-PAGE (Fig. S10*B*) showed that most of the purified protein remained as an intact F_OF_1 complex under these conditions, indicating preserved structural integrity needed for ATP synthase activity.

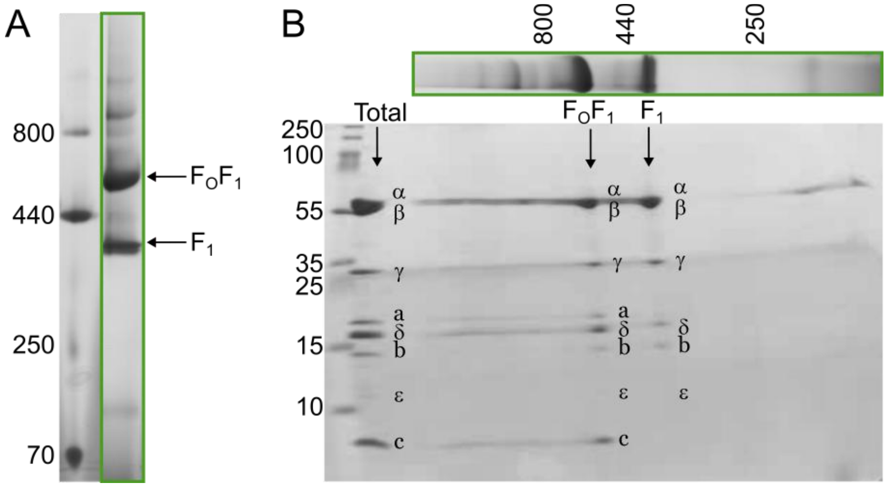

Fig. S10. (*A*) Blue native (BN)-PAGE of ATP synthase of the thermophilic *Bacillus* strain PS3. The main band was assigned to the intact protein complex with $M(F_OF_1)$ = 530 kDa, while a second band could be assigned to the water-soluble F1 subunit with $M(F_1)$ = 356 kDa (5). (*B*) The BN-PAGE bands were subsequently separated in a second dimension by SDS-PAGE. The resulting 2D-SDS-PAGE confirmed that all subunits were present in the main F_OF_1 band. In contrast, subunits a and c, which are specific to the F_O subunit, were absent in the F_1 subunit.

SI References

1. N. Kucerka, M. P. Nieh, J. Katsaras, Fluid phase lipid areas and bilayer thicknesses of commonly used phosphatidylcholines as a function of temperature. *Biochim. Biophys. Acta-Biomembranes* **1808**, 2761-2771 (2011).
2. M. Tutus *et al.*, Orientation-selective incorporation of transmembrane F_oF_1 ATP synthase complex from *Micrococcus luteus* in polymer-supported membranes. *Macromol. Biosci.* **8**, 1034-1043 (2008).
3. M. Schwamborn, J. Schumacher, J. Sibold, N. K. Teiwes, C. Steinem, Monitoring ATPase induced pH changes in single proteoliposomes with the lipid-coupled fluorophore Oregon Green 488. *Analyst* **142**, 2670-2677 (2017).
4. T. Suzuki, H. Ueno, N. Mitome, J. Suzuki, M. Yoshida, Fo of ATP synthase is a rotary proton channel: Obligatory coupling of proton translocation with rotation of c-subunit ring. *J. Biol. Chem*. **277**, 13281-13285 (2002).
5. Eiji Usukura et al., Torque generation and utilization in motor enzyme F0F1-ATP synthase. *J. Biol. Chem.* **287**, 1884-1891 (2012).
